# Prevalence and epidemiological distribution of selected foodborne pathogens in human and different environmental samples in Ethiopia: a systematic review and meta-analysis

**DOI:** 10.1186/s42522-021-00048-5

**Published:** 2021-09-03

**Authors:** Dinaol Belina, Yonas Hailu, Tesfaye Gobena, Tine Hald, Patrick Murigu Kamau Njage

**Affiliations:** 1 College of Veterinary Medicine, Haramaya University, P.O. Box 138, Dire Dawa, Ethiopia; 2 College of Agriculture and Environmental Sciences, Haramaya University, Dire Dawa, Ethiopia; 3 National Food Institute, Technical University of Denmark, Lyngby, Denmark; 4 College of Health and Medical Sciences, Haramaya University, Dire Dawa, Ethiopia

**Keywords:** Bacteria, FBP, Meta-analysis, Source attribution, Ethiopia

## Abstract

Bacterial Foodborne Pathogens (FBP) are the commonest cause of foodborne illness or foodborne diseases (FBD) worldwide. They contaminate food at any stages in the entire food chain, from farm to dining-table. Among these, the Diarrheagenic *Escherichia coli* (DEC), Non typhoidal *Salmonella* (NTS), *Shigella* spp. and *Campylobacter* spp. are responsible for a large proportion of illnesses, deaths; and, particularly, as causes of acute diarrheal diseases. Though existing studies indicate the problem may be severe in developing countries like Ethiopia, the evidence is commonly based on fragmented data from individual studies. A review of published and unpublished manuscripts was conducted to obtain information on major FBP and identify the gaps in tracking their source attributions at the human, animal and environmental interface. A total of 1753 articles were initially retrieved after restricting the study period to between January 2000 and July 2020. After the second screening, only 51 articles on the humans and 43 on the environmental sample based studies were included in this review. In the absence of subgroups, overall as well as human stool and environmental sample based pooled prevalence estimate of FBP were analyzed. Since, substantial heterogeneity is expected, we also performed a subgroup analyses for principal study variables to estimate pooled prevalence of FBP at different epidemiological settings in both sample sources. The overall random pooled prevalence estimate of FBP (*Salmonella,* pathogenic *Escherichia coli (E. coli), Shigella* and *Campylobacter* spp.) was 8%; 95% CI: 6.5–8.7, with statistically higher (*P* <  0.01) estimates in environmental samples (11%) than in human stool (6%). The subgroup analysis depicted that *Salmonella* and pathogenic *E. coli* contributed to 5.7% (95% CI: 4.7–6.8) and 11.6% (95% CI: 8.8–15.1) respectively, of the overall pooled prevalence estimates of FBD in Ethiopia. The result of meta-regression showed, administrative regional state, geographic area of the study, source of sample and categorized sample size all significantly contributed to the heterogeneity of *Salmonella* and pathogenic *E. coli* estimates. Besides, the multivariate meta- regression indicated the actual study year between 2011 and 2015 was significantly associated with the environmental sample-based prevalence estimates of these FBP. This systematic review and meta-analysis depicted FBP are important in Ethiopia though majority of the studies were conducted separately either in human, animal or environmental samples employing routine culture based diagnostic method. Thus, further FBD study at the human, animal and environmental interface employing advanced diagnostic methods is needed to investigate source attributions of FBD in one health approach.

**Supplementary Information:**

The online version contains supplementary material available at 10.1186/s42522-021-00048-5.

## Introduction

Foodborne pathogens (FBP) are biological agents like viruses, bacteria and parasites that can cause a foodborne illness or foodborne diseases (FBD). Foodborne disease (also referred to as foodborne illness or food poisoning) is any illness that results from the consumption of food contaminated with pathogenic bacteria, viruses, or parasites [1]. Foodborne illness or food poisoning is mostly resulted from eating contaminated, spoiled, or toxic food and basically, FBD can be due to foodborne infection, foodborne intoxication or foodborne intoxico-infection [2, 3].

Foodborne pathogens (FBP) cause millions of cases of sporadic illness and chronic complications, as well as large and challenging outbreaks in many countries and between countries [4]. The effect of these pathogens also varies from region to region as level of public awareness about food hygiene varies in different countries. Rane [5] and Paudyal et al. [6] explained, most of the FBP are introduced as exogenous contaminants during handling, processing and preparation rather than being present as endogenous contaminants. The problem is severe in developing countries like Ethiopia due to limitations in securing optimal hygienic food handling practices [7, 8].

Pathogenic bacteria contaminate food at any stages in the entire food chain from farm to dining-table [9]. Humans can acquire pathogens or their infections through consumption of a variety of contaminated foods and water, or through contact with infected livestock and other animal feces. Infected human and environment are also source of the infection [10, 11]. *Salmonella*, *Listeria monocytogenes*, *Escherichia coli* (*E. coli*), *Campylobacter* spp. [8, 12] and *Shigella* [7, 13] are among the most common FBP reported from Ethiopia. Specifically, FBP like Diarrheagenic *E. coli* and NTS are constantly being excreted into the environment in massive quantities and they are responsible for a large proportion of illnesses and deaths; more importantly, as sources of acute diarrheal diseases in children [14]. Food-producing animals are the major reservoirs for many FBP [4]. Direct and indirect contact with animals (livestock and other animal), and their feces carrying zoonotic pathogens or the farming environment are important risk factors for FBD. Hence, the health of people is connected to the health of animals and the environment [15].

In developing countries like Ethiopia, the primary sources of *Salmonella* infection in human are food animals such as cattle, poultry and swine, mainly via contamination of carcass with the gastrointestinal content during slaughtering [16, 17]. Cattle are also the major reservoirs of *E. coli* O157:H7 followed by sheep and goats. The pathogens are shed intermittently in feces of infected animals [18, 19]. *Escherichia coli* infection is usually severe in the old age and under five children; especially shiga toxin-producing *E. coli* O157: H7 causes the most severe condition in immune-compromised individuals and also in those healthy people exposed to a very high dose [2, 10]. Ethiopia is particularly vulnerable to the effect of zoonotic diseases because the economy is largely dependent on agriculture [20, 21] and roughly 80% of households have direct contact with domestic animals, creating an opportunity for infection and spread of disease [12, 22, 23]. A study conducted in Ethiopia reported high pathogenic *E. coli* prevalence up to 51.6% [24]. Tosisa [25] also reported, *E. coli* as one of the most common cause of acute infectious diarrhea in children. A recent meta-analysis of 30 articles from Ethiopia also showed 18.1% pooled prevalence estimates of *E. coli* in foods of animal origin [26].

A study conducted in Ethiopia reported 8.72, 5.68 and 1.08% pooled prevalence estimates of *Salmonella* in diarrheic children, adults and carriers, respectively. Nontyphi *Salmonella* accounted for 57.9% of the reported isolates [27]. Enteric *Salmonella* infection prevalence of 5.5% [24] and 1.3% [28], were also reported from Debre Berhan and Ambo, respectively in under five children (UFC). Eguale et al. [29] and Eguale et al. [17] also reported prevalence of 7.2, 4.7 and 4.4% *Salmonella* in human patients, poultry and swine respectively.

Even though bacterial FBP are important in Ethiopia; implementation of pathogen prevention and control intervention strategies are poor or challenging because there are no detailed surveillance and published data on the incidence of FBP [8]. Again, there is little well documented information regarding the current status, specifically on FBP pathotype detected, and the epidemiology and their source attributions in Ethiopia. Besides, a few systematic review and meta-analysis have been conducted on FBP in Ethiopia, and those reviews are only focused on FBP report in different food items, particularly in foods of animal origin, which did not consider the source of bacterial contamination. Thus, it is timely and pertinent to conduct a comprehensive scientific review on recent status of FBP in human, animal and other environmental samples to support proper and focused scholarly effort. Therefore, to the level of our knowledge, this is the first systematic review and meta-analysis report that investigates FBP in both human and environmental samples in the case of Ethiopia. Hence, this study aims at reviewing the reports available on major FBP and identifying the gaps in the source attributions of FBP of high importance (*Salmonella* and *E. coli*) at the human, animal and environmental interface.

## Methodology

Initially, a total of 2498 articles (2470 from PMC, BMC medicine and direct Google and 28 from university repositories) were searched for *Salmonella*, *E. coli, Shigella* and *Campylobacter* spp*.* However, to optimize the management of data, this study only focused on *Salmonella* and *E. coli*. The data extracted on *Shigella* and *Campylobacter* spp. is only used in the determination of the overall pooled prevalence estimates of FBP in Ethiopia (Table 4) and is not reported in this study. However, the data is accessible by requesting from the authors. On the other hand, most of the studies included in this analysis (fulfilling the inclusion criteria) reported only *E. coli* prevalence in general and did not mention specific pathotypes. Thus, in this study, if a sample is positive for any of the six *E. coli* pathotypes, it was considered as positive for pathogenic *E. coli,* and the *E. coli* mentioned refers to the pathogenic *E. coli*.

### Frame work of the study

For this systematic review and meta-analysis on FBP, we identified the analytical framework in which the public living in Ethiopia is considered as study population while FBP attribution and its epidemiology were taken as a phenomenon of interests. The context was the healthcare facilities and non-healthcare institutions involved in food establishments and veterinary institutes to where public health researchers usually give attentions. The review was framed based on research questions: What is the overall pooled prevalence of FBD, and which bacterial FBP are more important, in Ethiopia? Do the occurrences of *Salmonella* and *E. coli* vary in their epidemiological distribution, and what are their respective associated sources?

### Literature search strategy and pathogen prioritization

A comprehensive literature search was conducted electronically to collect published articles, short communications and study reports on FBP and its source attributions and epidemiology in Ethiopia. Published articles/or reports were searched from PMC and BMC (medicine) journal electronic data bases and also through direct Google search. Additionally, graduate thesis and dissertations were collected from University repositories like Addis Ababa and Haramaya Universities, and manual search was also conducted for unpublished manuscripts. In the searching process we used free text and medical subject heading terms combined with FBD/FBP related keywords. In the first steps, we selected specific key words (for supplementary file, see Annex 1) to search potential articles to identifying the most important FBP in children in Ethiopia.

By reviewing articles and reports searched by these terms, *Salmonella, E. coli, Shigella, campylobacter, Staphylococcus aureus* and *Listeria monocytogenes* were identified as the most prevalent FBP, particularly those associated with diarrhea in children in Ethiopia. Beside, we referred the work of Pieracci et al [12], who prioritized zoonotic diseases in Ethiopia using a one health approach, and reported that *Salmonella, Campylobacter* and *E. coli* are among the top eight bacterial FBP. Therefore, based on our preliminary review results (FBD in diarrheic children in Ethiopia), we decided to conduct the review on selected bacterial FBP i.e. *Salmonella, E. coli*, *Shigella* and *Campylobacter.* In this regard, the second searching steps were conducted to retrieve literatures for the identified four FBP or their disease conditions from the above mentioned databases and university repositories using different specific search terms combined with the genus name of the bacterial pathogen (Table 1). Studies published after the year 2000 whose abstracts were accessible as per the searching time were retrieved. The search was performed twice with the second search on July 24, 2020 after phase one aimed at checking for missed papers (if any).

**Table 1:** Search terms used to retrieve articles and number of articles searched for screening

Search terms developed and used	FBP				
	***Salmonella***		***E. coli***		Other source
	BMC	Pubmed	BMC	Pubmed	
“*Organism*” and “Ethiopia”	165	166	236	191	6
“*Organism*” and “Ethiopia” and “Diarrhea”	73	28	79	12	3
“*Organism*” and “Ethiopia” and “children” and “Diarrhea”	48	14	51	6	1
“*Organism*” and “Ethiopia” and “under five children” and “Diarrhea”	40	6	39	2	4
“Prevalence” and “*Organism*” and “Ethiopia” and “Diarrhea”	53	19	55	4	1
“Isolation” and “*Organism*” and “Ethiopia” and “children” and “Diarrhea”	22	10	26	3	2
“Epidemiology” and “*Organism”* and “Ethiopia” and “children”	29	19	37	12	0
“Prevalence” and “*Organism*” and “Ethiopia” and “children” and “Diarrhea”	41	11	42	3	0
“Diarrheagenic *E. coli*” and “Ethiopia”			0	192	2
Total	**471**	**273**	**565**	**425**	**19**

N. B.: The word “organism” separately replaced by *Salmonella* and *E. coli*

Other source *=* unpublished sources like university repositories and direct Google search

### Eligibility criteria and screening procedures

#### Inclusion criteria: study area

Only studies conducted in Ethiopia. Population: Studies including metrics for sample size and which directly and/or indirectly provided prevalence of FBD/FBP with or without age specific estimates were included. Study design: All observational and retrospective studies as well as baseline investigations from prospective studies with defined FBD/FBP in which the actual study period was limited to within the last 20 years. Sample type: only studies which reported FBP prevalence in stool samples (in human case) and environmental samples were included. Language: Only articles reported in English language were considered. Period and publication condition: Both published and unpublished articles in which the actual data collection, processing and laboratory analysis were restricted to the period from January 2000 to July 2020 were included.

#### Exclusion criteria

Article citations with no abstracts and/or full texts, duplicate studies, and studies in which number of positive cases and total sample sizes were not reported (if the prevalence was only reported as percentage) were excluded. Studies in which, general FBD burden is reported (in which the pathogen is not specified), the diagnostic method (microbial detection technique) is not described and FBP studies in wildlife, were also excluded from the meta-analysis.

### Screening procedures and relevance of the study

Regarding relevance and quality of the studies, two independent reviewers (DB and YH) first identified both the details of the study variables or outcomes (Fig. 2) and the search terms based on research questions under the framework. Search terms and screening methods were modified with justification to include hand searches through discussion with TG. Then the other authors TH and PMK, commented on the overall framework and the data synthesis approaches. Predefined guidelines for accurate and transparent health estimates reporting (GATHER) checklist was also used for screening. In addition, in order to minimize biases, we agreed to include publication year together with actual study year as well as to extend year of study to 20 years which in turn broaden the number of included studies (i.e., data from January 2000 to July 2020 studies).

**Fig. 1:**
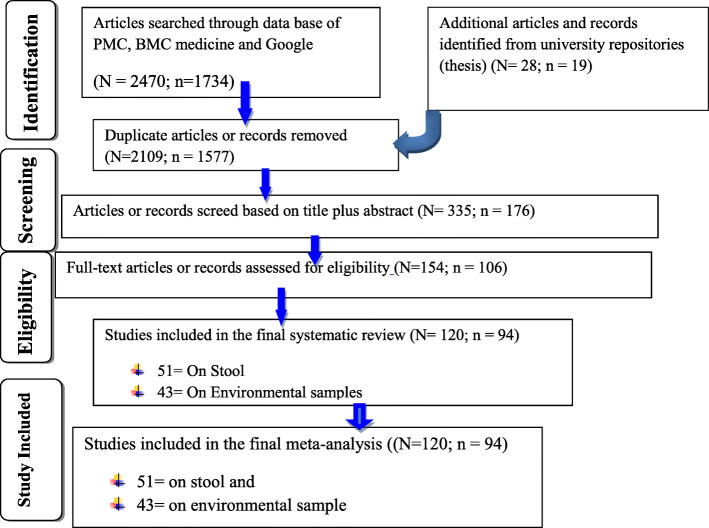
Flow chart of study selection for systematic review and meta-analysis of *Salmonella* and pathogenic *E. coli* in Ethiopia**.** Note: N = number of manuscripts used for overall pooled prevalence estimates of FBD; n = number of studies specific to *Salmonella* and pathogenic *E. coli* used in the current FBP analysis

Then two independent reviewers (DB and YH) searched and screened out articles using titles and abstracts. De-duplication of the studies was performed using endnote software which was also corrected by manual method. Relevance of the studies was checked and references were excluded up on the two reviewers’ agreement. Also dissents raised at this first screening stage were resolved by involvement of the other authors. Each complete article was screened separately by two reviewers per reference for the inclusion and exclusion criteria developed prior to data extraction. Any conflict raised at this second screening stage was also resolved with the third reviewer prior to data extraction. Relevant data/information related to study characteristics were assessed from the retrieved abstracts and full articles, and the studies that fail to fit the study criteria were removed. Thus, articles used in our study passed through different screening steps from identification to final article inclusion. Of the 1753 manuscripts searched, only 94 studies were found eligible for systematic review and meta-analysis (Table 1; Fig. 1) though 120 studies screed from 2498 searched manuscripts were used for the overall pooled prevalence estimates of FBD (Fig. 1).

### Measurement of the outcome variables and their description

We have two main outcomes, namely, the human stool, and environmental sample based outcome variables of FBP, because some of the studies focused on FBP in human stool in different age groups, and other studies were conducted on different environmental samples. Some of the studies were also conducted before ten years and others were relatively recent, and etc. So it is possible that these studies would yield different summary estimates. In fact, a thorough moderator analysis is more informative than a single estimate of summary effect size when meta-analytic data being examined contains substantial heterogeneity [30]. Moderators are often categorical, either because of inherent factors or because the information provided in articles does not allow for more fine-grained coding [31].

Similar to primary studies, moderator analyses have a sample of participants (i.e., the studies included in a meta-analysis), one or multiple independent variables (i.e., moderating variables) and one dependent variable (i.e., effect sizes within each subgroup) [30]. According to Hamza et al. [32], under the framework of subgroup analysis, the total set of studies is split into two or more subgroups based on the categories within a categorical moderator and the effect in one subgroup of studies is compared with that in the rest of the subgroup(s) of studies. When the between-study variance (*I*^*2*^) is greater than zero (0%), the overall heterogeneity can be accounted for by the true differences between studies. Thus, it makes sense to apply sub-group analyses or meta-regressions to identify potential moderating factors that can explain the inconsistencies between effect sizes across studies or factors that can influence or explain the relationships [30, 33]. It is assumed that an I^2^ of 25, 50, and 75% indicate low, medium, and large heterogeneity, respectively [34, 35].

**Fig. 2:**
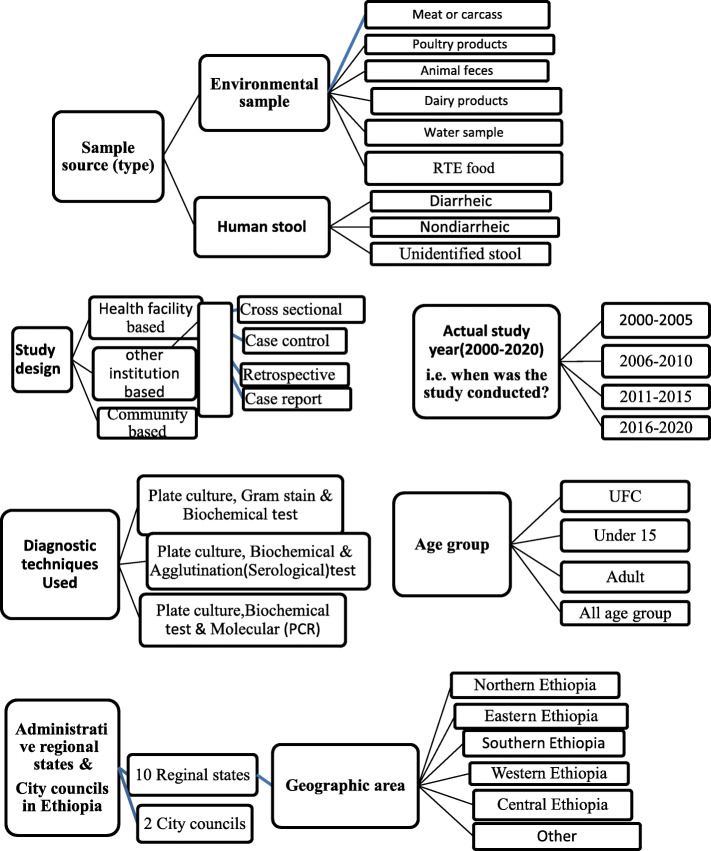
Details of the study variables used to estimate FBP source attribution

Hence, in this study, the overall and separate subgroup prevalence estimates of FBP with their epidemiological distribution and the source attributions were calculated for both human stool and environmental sample based outcomes. Important variables including age group, sample type, diagnostic techniques, study design or type, actual study year (Fig. 2) were considered in order to determine prevalence estimates of FBD and the epidemiological risk factors. Of the nine administrative regional states (the 10th region, recently established region, Sidama is considered in SNNP) and two city councils in Ethiopia, overall studies conducted in eight regions (only study from Afar region is not screened) and the two city councils were included.

Likewise, to test how the summary effects would change with different types of studies or different characteristics of participants in the study, we need to obtain at least the minimum acceptable number of studies in order to run subgroup analyses and meta-regression [33]. We also followed the principle that when the sizes of the included studies are moderate or large, there should be at least 6 to 10 studies for a continuous study level variable; and for a (categorical) subgroup variable, each subgroup should have a minimum of 4 studies [36].

Of the 11 subgroup variables analysed in the present study, majority of them encompass more than 10 studies. Variables with less than 4 included studies were either excluded (e.g. molecular diagnostic technique) or merged and named with new designation to be used for pooled estimate comparison. For instance, few study areas were merged mainly based on their proximity in geographic area plus number of studies, and then labeled as “other”. Likewise, the studies conducted in Dire Dawa city and Harari regional state were less than the minimum limits of subgroup analysis criteria and, hence, we merged them as Dawa and Harari regional state. Regarding environmental samples, feces from any domestic animal expect that of poultry was designated as “animal feces” in the subgroup analysis.

On the other hand, in the systematic review, comparison of study variables were made regardless of the number of studies included in each variable. Here, we only made technical term modification and arrangements in the review results presentation. For instance, the variable “others” in study area, represents a study in which a laboratory sample was collected from two or more different regions, geographic or study areas (Tables 2 and 3). Besides, some of the articles included in this review reported prevalence of FBP in either carcass directly collected from abattoirs or meat samples collected from butcher shops and hotels. Few of the articles regarded both abattoir and butcher shop samples as a carcass. In this review, however, the term “retail meat” for carcass and its contact surface samples collected from butcher shops, and for the corresponding samples from abattoir “*abattoir carcass*” were used to estimate specific FBD source attribution. Moreover, the term *food (large) animal* (e.g. food animal feces) was used in place of either pooled sample (from different domestic animals as a single) or individually collected from cattle, sheep, goat, camel and pig (swine). “*Unidentified stool*” is also used for cases in which the condition of stool collected for laboratory analysis was not specifically stated in the screened articles, or in cases when both “diarrheic” and “non-diarrheic” samples were analyzed in as a single (one) result.

**Table 2:** Summary of eligible studies of FBD and variables used for prevalence estimates of pathogens in human stool samples in Ethiopia

FBP	Reference	Year Of Study	Geographic area	Region	District or town	Study design	Age group	Stool condition	Diagnostic technique	Sample size	No positive	95%-CI %
*Eschechia coli*	[37]	2014	Central Ethiopia	other	other	HFB CS	All age	Diarrheic	C, Gs and BT	70	0	0.00(0.04–10.27)
*Eschechia coli*	[38]	2017	Northwest Ethiopia	Amhara	Bahir Dar	HFB CS	UFC	Diarrheic	C, Gs and BT	344	35	10.17(7.39–13.84)
*Eschechia coli*	[39]	2012	Northwest Ethiopia	Amhara	Bahir Dar	HFB CS	UFC	Diarrheic	C, BT and Ag	422	204	48.34(43.60–53.11)
*Eschechia coli*	[40]	2015	Southern Ethiopia	SNNP	Hawassa	HFB retro	All age	Diarrheic	C, Gs and BT	170	0	0.00(0.02–4.50)
*Eschechia coli*	[41]	2017	Central Ethiopia	A.A	A.A	HFB CS	under 15	Diarrheic	C, Gs and BT	290	13	4.48(2.62–7.57)
*Eschechia coli*	[41]	2017	Central Ethiopia	A.A	A.A	HFB CS	UFC	Diarrheic	C, Gs and BT	140	8	5.71(2.88–11.01)
*Eschechia coli*	[42]	2015	Central Ethiopia	A.A	A.A	HFB CS	UFC	Diarrheic	C, Gs and BT	253	61	24.11(19.24–29.76)
*Eschechia coli*	[43]	2007	Northwest Ethiopia	Amhara	Gondar	HFB CS	All age	Diarrheic	C, BT and Ag	384	0	0.00(0.01–2.04)
*Eschechia coli*	[44]	2016	Southern Ethiopia	SNNP	Hawassa	HFB CS	Adult	Diarrheic	C, BT and Ag	102	2	1.96(0.49–7.50)
*Eschechia coli*	[44]	2016	Southern Ethiopia	SNNP	Hawassa	HFB CS	Adult	Diarrheic	C, BT and Ag	113	0	0.00(0.03–6.62)
*Eschechia coli*	[45]	2013	Central Ethiopia	A.A	A.A	HFB CS	UFC	Diarrheic	C, Gs and BT	253	61	24.11(19.24–29.76)
*Eschechia coli*	[46]	2015	Eastern Ethiopia	Dire dawa	Dire dawa	HFB CS	UFC	Diarrheic	C, Gs and BT	196	25	12.76(8.77–18.20)
*Eschechia coli*	[24]	2016	Central Ethiopia	Amhara	D/ Berhan	HFB CS	UFC	Diarrheic	C, Gs and BT	163	47	28.83(22.40–36.25)
*Eschechia coli*	[47]	2018	Northwest Ethiopia	Amhara	Gondar	IB CS	Adult	Nondiarrheic	C, Gs and BT	257	5	1.95(0.81–4.59)
*Eschechia coli*	[48]	2015	Northwest Ethiopia	Amhara	D/Markos	HFB retro	UFC	Unidentified	C, Gs and BT	50	1	2.00(1.95–17.02)
*Eschechia coli*	[48]	2015	Northwest Ethiopia	Amhara	D/Markos	HFB retro	All age	Unidentified	C, Gs and BT	58	1	1.72(0.24–11.24)
*Salmonella*	[29]	2014	Central Ethiopia	A.A	A.A	HFB CS	All age	Diarrheic	C, BT, Ag and Mol	957	59	6.17(4.81–7.88)
*Salmonella*	[49]	2016	Central Ethiopia	A.A	A.A	HFB CS	All age	Diarrheic	C, BT and Ag	99	7	7.07(3.41–14.10)
*Salmonella*	[50]	2017	Southern Ethiopia	SNNP	Hosanna	HFB CS	UFC	Diarrheic	C, Gs and BT	204	2	0.98(0.25–3.83)
*Salmonella*	[38]	2017	Northwest Ethiopia	Amhara	Bahir Dar	HFB CS	UFC	Diarrheic	C, Gs and BT	344	6	1.74(0.79–3.83)
*Salmonella*	[51]	2012	Northwest Ethiopia	Amhara	Bahir Dar	HFB CS	UFC	Diarrheic	C, BT and Ag	422	33	7.82(5.61–10.80)
*Salmonella*	[52]	2017	Southern Ethiopia	SNNP	A/Minch	HFB CS	UFC	Diarrheic	C, Gs and BT	167	21	12.57(8.34–18.52)
*Salmonella*	[40]	2015	Southern Ethiopia	SNNP	Hawassa	HFB retro	All age	Diarrheic	C, Gs and BT	170	5	2.94(1.23–6.87)
*Salmonella*	[53]	2002	Central Ethiopia	A.A	A.A	HFB CS	All age	Diarrheic	C, Gs and BT	205	22	10.73(7.17–15.76)
*Salmonella*	[54]	2016	Southeast Ethiopia	Oromia	Bale Robe	HFB CS	UFC	Diarrheic	C, Gs and BT	139	7	5.04(2.42–10.19)
*Salmonella*	[54]	2016	Southeast Ethiopia	Oromia	Goba	HFB CS	UFC	Diarrheic	C, Gs and BT	283	22	7.77(5.17–11.52)
*Salmonella*	[55]	2001	Southwest Ethiopia	Oromia	Jimma	HFB CS	Adult	Diarrheic	C, Gs and BT	152	11	7.24(4.05–12.59)
*Salmonella*	[41]	2017	Central Ethiopia	A.A	A.A	HFB CS	UFC	Diarrheic	C, Gs and BT	140	4	2.86(1.08–7.36)
*Salmonella*	[41]	2017	Central Ethiopia	A.A	A.A	HFB CS	under 15	Diarrheic	C, Gs and BT	290	7	2.41(1.16–4.98)
*Salmonella*	[56]	2003	Southwest Ethiopia	Oromia	Jimma	HFB CS	under 15	Diarrheic	C, Gs and BT	430	21	4.88(3.21–7.37)
*Salmonella*	[57]	2012	Southwest Ethiopia	Oromia	Jimma	HFB CS	UFC	Diarrheic	C, Gs and BT	179	12	6.70(3.85–11.43)
*Salmonella*	[57]	2012	Southwest Ethiopia	Oromia	Jimma	HFB CS	under 15	Diarrheic	C, Gs and BT	260	16	6.15(3.80–9.81)
*Salmonella*	[58]	2014	Northwest Ethiopia	Amhara	Gondar	HFB CS	All age	Diarrheic	C, Gs and BT	372	4	1.08(0.40–2.83)
*Salmonella*	[29]	2014	Central Ethiopia	A.A	A.A	HFB CS	UFC	Diarrheic	C, BT, Ag and Mol	160	10	6.25(3.40–11.23)
*Salmonella*	[13]	2016	Northwest Ethiopia	Amhara	Wegera	CB CS	UFc	Diarrheic	C, Gs and BT	112	1	0.89(0.13–6.06)
*Salmonella*	[59]	2012	Northern Ethiopia	Tigray	Mekele	HFB CS	under 15	Diarrheic	C, BT and Ag	260	19	7.31(4.71–11.17)
*Salmonella*	[59]	2012	Northern Ethiopia	Tigray	Mekele	HFB CS	UFC	Diarrheic	C, BT and Ag	115	14	12.17(7.34–19.51)
*Salmonella*	[42]	2015	Central Ethiopia	A.A	A.A	HFB CS	UFC	Diarrheic	C, Gs and BT	253	10	3.95(2.14–7.19)
*Salmonella*	[60]	2015	Central Ethiopia	A.A	A.A	HFB CS	under 10	Diarrheic	C, Gs and BT	22	0	0.00(0.13–26.81)
*Salmonella*	[61]	2019	Southern Ethiopia	SNNP	Hawassa	HFB CS	under 15	Diarrheic	C, Gs and BT	263	1	0.38(0.05–2.65)
*Salmonella*	[43]	2007	Northwest Ethiopia	Amhara	Gondar	HFB CS	All age	Diarrheic	C, Gs and BT	384	6	1.56(0.70–3.43)
*Salmonella*	[44]	2016	Southern Ethiopia	SNNP	Hawassa	HFB CS	Adult	Diarrheic	C, BT and Ag	102	7	6.86(3.31–13.70)
*Salmonella*	[62]	2014	Southwest Ethiopia	Oromia	Jimma	HFB CS	All age	Diarrheic	C, Gs and BT	176	19	10.80(6.99–16.30)
*Salmonella*	[62]	2014	Southwest Ethiopia	Oromia	Jimma	HFB CS	under 10	Diarrheic	C, Gs and BT	54	6	11.11(5.08–22.60)
*Salmonella*	[45]	2013	Central Ethiopia	A.A	A.A	HFB CS	UFC	Diarrheic	C, Gs and BT	253	10	3.95(2.14–7.19)
*Salmonella*	[46]	2015	Eastern Ethiopia	Dire dawa	Dire dawa	HFB CS	UFC	Diarrheic	C, Gs and BT	196	7	3.57(1.71–7.30)
*Salmonella*	[63]	2017	Western Ethiopia	Gambella	Gambella	IB CS	UFC	Diarrheic	C, Gs and BT	134	4	2.99(1.12–7.68)
*Salmonella*	[64]	2012	Central Ethiopia	SNNP	Butajira	HFB CS	under 15	Diarrheic	C, BT and Ag	174	22	12.64(8.47–18.46)
*Salmonella*	[64]	2012	Central Ethiopia	SNNP	Butajira	HFB CS	Adult	Diarrheic	C, BT and Ag	208	18	8.65(5.52–13.32)
*Salmonella*	[65]	2011	Southern Ethiopia	SNNP	Hawassa	HFB CS	UFC	Diarrheic	C, BT and Ag	158	4	2.53(0.95–6.55)
*Salmonella*	[66]	2007	Eastern Ethiopia	Harari	Harar	HFB CS	Adult	Diarrheic	C, Gs and BT	244	28	11.48(8.04–16.12)
*Salmonella*	[67]	2016	Western Ethiopia	Oromia	Nekemt	HFB CS	All age	Diarrheic	C, Gs and BT	422	30	7.11(5.01–9.99)
*Salmonella*	[68]	2017	Central Ethiopia	Oromia	Adama	HFB CS	All age	Diarrheic	C, BT and Ag	232	20	8.62(5.63–12.98)
*Salmonella*	[28]	2014	Central Ethiopia	Oromia	Ambo	HFB CS	UFC	Diarrheic	C, BT and Ag	239	3	1.26(0.41–3.82)
*Salmonella*	[69]	2012	Northwest Ethiopia	Amhara	Bahir Dar	HFB CS	UFC	Diarrheic	C, BT and Ag	422	33	7.82(5.61–10.80)
*Salmonella*	[24]	2016	Central Ethiopia	Amhara	D/ Berhan	HFB CS	UFC	Diarrheic	C, Gs and BT	163	5	3.07(1.28–7.16)
*Salmonella*	[70]	2009	Northwest Ethiopia	Amhara	Bahir Dar	IB CS	Adult	Mixed	C, Gs and BT	384	6	1.56(0.70–3.43)
*Salmonella*	[71]	2006	Southwest Ethiopia	Oromia	Jimma	HFB CS	under 15	Mixed	C, BT and Ag	400	10	2.50(1.35–4.58)
*Salmonella*	[71]	2006	Central Ethiopia	A.A	A.A	HFB CS	under 15	Mixed	C, BT and Ag	825	55	6.67(5.15–8.58)
*Salmonella*	[72]	2016	Northwest Ethiopia	Amhara	D/Markos	IB CS	Adult	Nondiarrheic	C, Gs and BT	220	8	3.64(1.83–7.10)
*Salmonella*	[73]	2013	Central Ethiopia	A.A	A.A	IB CS	Adult	Nondiarrheic	C, Gs and BT	172	6	3.49(1.58–7.55)
*Salmonella*	[74]	2018	Southern Ethiopia	SNNP	Hawassa	IB CS	Adult	Nondiarrheic	C, Gs and BT	236	5	2.12(0.88–4.99)
*Salmonella*	[55]	2001	Southwest Ethiopia	Oromia	Jimma	HFB CS	Adult	Nondiarrheic	C, Gs and BT	220	0	0.00(0.01–3.51)
*Salmonella*	[75]	2017	Southern Ethiopia	SNNP	Wolkite	IB CS	Adult	Nondiarrheic	C, Gs and BT	170	8	4.71(2.37–9.13)
*Salmonella*	[13]	2016	Northwest Ethiopia	Amhara	Wegera	CB CS	UFC	Nondiarrheic	C, Gs and BT	113	1	0.88(0.12–6.01)
*Salmonella*	[47]	2018	Northwest Ethiopia	Amhara	Gondar	IB CS	Adult	Nondiarrheic	C, Gs and BT	257	3	1.17(0.38–3.56)
*Salmonella*	[44]	2016	Southern Ethiopia	SNNP	Hawassa	HFB CS	Adult	Nondiarrheic	C, BT and Ag	113	4	3.54(1.33–9.05)
*Salmonella*	[76]	2015	Southern Ethiopia	SNNP	A/ Minch	IB CS	Adult	Nondiarrheic	C, Gs and BT	345	24	6.96(4.71–10.17)
*Salmonella*	[77]	2016	Eastern Ethiopia	Harari	Harar	IB CS	Adult	Nondiarrheic	C, Gs and BT	417	15	3.60(2.18–5.88)
*Salmonella*	[63]	2017	Western Ethiopia	Gambella	Gambella	IB CS	UFC	Nondiarrheic	C, Gs and BT	134	2	1.49(0.37–5.77)
*Salmonella*	[78]	2017	Southern Ethiopia	SNNP	Sodo	IB CS	Adult	Nondiarrheic	C, BT and Ag	387	35	9.04(6.56–12.34)
*Salmonella*	[79]	2017	Eastern Ethiopia	Dire dawa	Dire dawa	CB CS	Adult	Nondiarrheic	C, Gs and BT	218	13	5.96(3.49–10.00)
*Salmonella*	[80]	2016	Southwest Ethiopia	oromia	jimma	IB CS	Adult	Nondiarrheic	C, Gs and BT	50	9	18.00(9.64–31.11)
*Salmonella*	[81]	2015	Central Ethiopia	A.A	A.A	HFB retro	All age	Unidentified	C, Gs and BT	136	43	31.62(24.36–39.89)
*Salmonella*	[48]	2015	Northwest Ethiopia	Amhara	D/Markos	HFB retro	UFC	Unidentified	C, Gs and BT	50	12	24.00(14.16–37.67)
*Salmonella*	[48]	2015	Northwest Ethiopia	Amhara	D/Markos	HFB retro	All age	Unidentified	C, Gs and BT	58	24	41.38(29.51–54.34)

*A.A* Addis Ababa, *C* Culture (plate culture), *Gs* Gram stain, *BT* Biochemical test, *Ag* Antigen detection (agglutination), *Mol* Molecular like PCR, *HFB CS* Health facility based cross-sectional study, *CB Cs-s* Community based cross-sectional study, *HFB retro* Health facility based retrospective study, *IB CS* Institution based cross-sectional study, *UFC* under five children, *SNNP* Southern Nations, Nationalities, and Peoples

**Table 3:** Summary of eligible studies of FBP and variables used for prevalence estimates of pathogens in different environmental samples in Ethiopia

FBP	Author	Year of study	Region	Geographic area	Study area	Sample type	Sample size	No positive	95%-CI %
*E. coli*	[82]	2013	Central Eth	Oromia	Mojo	Abattoir carcass	144	4	2.78(1.05–7.17)
*E. coli*	[83]	2012	Central Eth	AA	A.A	Abattoir carcass	192	11	5.73(3.20–10.05)
*E. coli*	[84]	2016	Central Eth	Others	Other	Abattoir carcass	219	36	16.44(12.10–21.95)
*E. coli*	[85]	2015	Central Eth	Others	Other	Abattoir carcass	635	82	12.91(10.52–15.75)
*E. coli*	[86]	2014	Eastern Eth	Somali	Somali	Abattoir carcass	93	3	3.23(1.04–9.53)
*E. coli*	[87]	2014	Eastern Eth	Somali	Jigjiga	Abattoir carcass	70	2	2.86(0.72–10.71)
*E. coli*	[19]	2015	Southern Eth	SNNP	Hawassa	Abattoir carcass	150	4	2.67(1.00–6.89)
*E. coli*	[37]	2014	Central Eth	Others	Other	Abattoir carcass environmental	1247	6	0.48(0.22–1.07)
*E. coli*	[82]	2013	Central Eth	Oromia	Mojo	Abattoir carcass environmental	228	16	7.02(4.34–11.15)
*E. coli*	[86]	2014	Eastern Eth	Somali	Somali	Abattoir carcass environmental	142	3	2.11(0.68–6.34)
*E. coli*	[19]	2015	Southern Eth	SNNP	Hawassa	Abattoir carcass environmental	240	7	2.92(1.40–5.99)
*E. coli*	[37]	2014	Central Eth	Others	Other	Carcass	865	3	0.35(0.11–1.07)
*E. coli*	[88]	2010	Northern Eth	Tigray	Mekelle	Carcass	100	9	9.00(4.75–16.40)
*E. coli*	[84]	2016	Central Eth	Others	Other	Chicken viscera or meat	73	27	36.99(26.74–48.56)
*E. coli*	[89]	2016	Central Eth	Oromia	Ambo	Chicken viscera or meat	191	62	32.46(26.20–39.42)
*E. coli*	[90]	2018	Central Eth	Oromia	Adami tulu	Dairy farm environment	254	27	10.63(7.39–15.06)
*E. coli*	[91]	2017	Central Eth	Oromia	Bishoftu	Dairy products	135	31	22.96(16.64–30.80)
*E. coli*	[92]	2011	Northwest Eth	Amhara	Gondar	Dairy products	107	16	14.95(9.37–23.02)
*E. coli*	[93]	2011	Others	Oromia	Other	Dairy products	53	24	45.28(32.52–58.70)
*E. coli*	[94]	2018	Northern Eth	Amhara	S/ wollo	Fish and contact surfaces	410	6	1.46(0.66–3.22)
*E. coli*	[37]	2014	Central Eth	Others	Other	Food animal feces	370	7	1.89(0.90–3.91)
*E. coli*	[95]	2013	Northern Eth	Amhara	Kombolcha	Food animal feces	201	74	36.82(30.43–43.70)
*E. coli*	[96]	2017	Northern Eth	Amhara	S/ wollo	Food animal feces	123	100	81.30(73.43–87.25)
*E. coli*	[19]	2015	Southern Eth	SNNP	Hawassa	Food animal feces	150	7	4.67(2.24–9.46)
*E. coli*	[97]	2017	Northern Eth	Tigray	Mekelle	Hand contacting surfaces	300	8	2.67(1.34–5.24)
*E. coli*	[98]	2018	Eastern Eth	Oromia	G/bordode	Milk container and milkers	60	21	35.00(24.06–47.79)
*E. coli*	[99]	2017	Northwest Eth	Amhara	Gondar	Other RTE food	72	15	20.83(12.97–31.73)
*E. coli*	[100]	2012	Northwest Eth	Amhara	Bahir dar	Other RTE food	40	29	72.50(56.84–84.07)
*E. coli*	[101]	2018	Southern Eth	SNNP	A/minch	Other RTE food	347	109	31.41(26.74–36.49)
*E. coli*	[102]	2015	Western Eth	Benishangul	Asossa	Raw milk	380	129	33.95(29.4–38.9)
*E. coli*	[90]	2018	Central Eth	Oromia	Adami tulu	Raw milk	154	15	9.74(5.96–15.53)
*E. coli*	[103]	2015	Eastern Eth	Somali	Fafen	Raw milk	126	34	26.98(19.96–35.39)
*E. coli*	[94]	2018	Eastern Eth	Oromia	G/bordode	Raw milk	141	64	45.39(37.37–53.66)
*E. coli*	[104]	2014	Eastern Eth	Somali	Jigjiga	Raw milk	120	70	58.33(49.34–66.81)
*E. coli*	[105]	2017	Northern Eth	Tigray	Mekelle	Raw milk	315	67	21.27(17.10–26.14)
*E. coli*	[37]	2014	Central Eth	Others	Other	Retrial meat	150	1	0.67(0.09–4.58)
*E. coli*	[91]	2017	Central Eth	Oromia	Bishoftu	Retrial meat	65	9	13.85(7.36–24.52)
*E. coli*	[83]	2012	Central Eth	AA	A.A	Retrial meat	192	28	14.58(10.26–20.31)
*E. coli*	[106]	2014	Central Eth	Others	Other	Retrial meat	195	5	2.56(1.07–6.01)
*E. coli*	[87]	2014	Eastern Eth	Somali	Jigjiga	Retrial meat	70	4	5.71(2.16–14.26)
*E. coli*	[19]	2015	Southern Eth	SNNP	Hawassa	Retrial meat	150	3	2.00(0.65–6.02)
*E. coli*	[107]	2016	Southwest Eth	Oromia	Jimma	Retrial meat	88	21	23.86(16.11–33.85)
*E. coli*	[108]	2009	Southwest Eth	Oromia	Jimma	Retrial meat and contact surfaces	165	44	26.67(20.48–33.93)
*E. coli*	[37]	2014	Central Eth	Others	Other	Retrial meat contact surfaces	570	2	0.35(0.09–1.39)
*E. coli*	[106]	2014	Central Eth	Others	Other	Retrial meat contact surfaces	330	4	1.21(0.46–3.18)
*E. coli*	[19]	2015	Southern Eth	SNNP	Hawassa	Retrial meat contact surfaces	90	1	1.11(0.16–7.46)
*E. coli*	[107]	2016	Southwest Eth	Oromia	Jimma	Retrial meat contact surfaces	80	14	17.50(10.65–27.41)
*E. coli*	[109]	2017	Central Eth	AA	A.A	River water	94	23	24.47(16.83–34.14)
*E. coli*	[93]	2011	Others	Oromia	Other	River water	233	128	54.94(48.50–61.21)
*E. coli*	[110]	2012	Northwest Eth	Amhara	Gondar	Wastewater	113	13	11.50(6.80–18.81)
*Salmonella*	[111]	2015	Central Eth	AA	A.A	Abattoir carcass	280	13	4.64(2.71–7.83)
*Salmonella*	[112]	2015	Central Eth	AA	A.A	Abattoir carcass	159	4	2.52(0.95–6.51)
*Salmonella*	[80]	2016	Southwest Eth	Oromia	Jimma	Abattoir carcass	195	22	11.28(7.54–16.54)
*Salmonella*	[113]	2008	Central Eth	Oromia	Mojo	Abattoir carcass	606	44	7.26(5.45–9.62)
*Salmonella*	[80]	2016	Southwest Eth	Oromia	Jimma	Abattoir carcass environmental	245	20	8.16(5.33–12.31)
*Salmonella*	[113]	2008	Central Eth	Oromia	Mojo	Abattoir carcass environmental	634	45	7.10(5.34–9.38)
*Salmonella*	[114]	2015	Northwest Eth	Amhara	Gondar	Dairy products	165	3	1.59(0.51,4.80)
*Salmonella*	[115]	2015	Central Eth	AA	A.A	Dog feces	360	42	11.67(8.74–15.41)
*Salmonella*	[116]	2015	Others	Others	Other	Drinking water	222	0	0.00(0.01–3.48)
*Salmonell*a	[117]	2012	Southwest Eth	Oromia	Jimma	Drinking water	90	3	3.33(1.08–9.83)
*Salmonella*	[118]	2013	Central Eth	AA	A.A	Food animal feces	1203	30	2.49(1.75–3.54)
*Salmonella*	[112]	2015	Central Eth	AA	A.A	Food animal feces	567	23	4.06(2.71–6.03)
*Salmonella*	[119]	2013	Southwest Eth	Oromia	Jimma	Hand contacting surface	100	10	10.00(5.47–17.60)
*Salmonella*	[114]	2015	Northwest Eth	Amhara	Gondar	Meat or carcass	110	9	8.18 (4.31–14.98)
*Salmonella*	[101]	2018	Southern Eth	SNNP	A/minch	Other RTE food	347	46	13.26(10.08–17.25)
*Salmonella*	[100]	2012	Northwest Eth	Amhara	Bahir dar	Other RTE food	40	23	57.50(41.96–71.69)
*Salmonella*	[120]	2015	Southern Eth	SNNP	Other	Poultry and contact surfaces	270	45	16.67(12.68–21.60)
*Salmonella*	[121]	2018	Southwest Eth	Oromia	Jimma	Poultry and contact surfaces	415	11	2.65(1.47–4.72)
*Salmonella*	[17]	2014	Central Eth	AA	A.A	Poultry feces	549	26	4.74(3.24–6.86)
*Salmonella*	[114]	2015	Northwest Eth	Amhara	Gondar	Poultry products	85	9	10.59 (5.60–19.11)
*Salmonella*	[103]	2015	Eastern Eth	Somali	Fafen	Raw milk	126	19	15.08(9.83–22.44)
*Salmonella*	[104]	2014	Eastern Eth	Somali	Jigjiga	Raw milk	120	4	3.33(1.26–8.54)
Salmonella	[122]	2013	Northwest Eth	Amhara	Gondar	Retrial meat	90	32	35.56(26.38–45.93)
*Salmonella*	[104]	2009	Southwest Eth	Oromia	Jimma	Retrial meat and contact surfaces	165	2	1.21(0.30–4.71)
*Salmonella*	[122]	2013	Northwest Eth	Amhara	Gondar	Retrial meat contact surfaces	216	21	9.72(6.42–14.45)

In this analysis, the prevalence estimates were taken from the random-effect model results. Because, the total variance of a study is the summary of the between and within-study variance and is used to assign weights under the random-effects model. In the absence of subgroups, the estimate of between-study variance (*τ*^2^) is computed based on the dispersion of all studies from the grand mean [34]. According to Borenstein [34], in the random-effects subgroup analysis, *R*^2^ index (explainable proportion of the between-study variance) can be employed in meta-regression to indicate the proportion of true heterogeneity across all studies that can be accounted for by one or a set of moderators in order to quantify the magnitude of their impact on study effects. Basically the R^2^ value ranges between 0(0%) and 1(100%).

### Data extraction

Following the second screening stage, standardized data abstraction format was prepared in Microsoft excel and important data related to study characteristics (Table 2) was extracted from included articles independently by the first two authors. Information such as: name of the first author, sample size, number of positive samples, actual study year, year of publication, participant age group, sample type, diagnostic techniques, study design employed, administrative regional state or city council, geographic area, specific study area (district or town) and bacterial pathogen isolated, were all extracted and considered in the analysis. Finally, the authors independently made cross-checks for the extracted information before the actual process of data analysis.

### Data analysis and interpretation

Data management was initially performed on the data file stored in the abstraction format to prepare a comma separated values (.csv) file for further analysis. For the systematic review, prevalence of FBP and its 95% confidence interval (Tables 2 and 3) was calculated for each included study, from the extracted sample size and the number of positive samples. This was because in some of the screened articles, the FBP was reported only with sample size and number of positive samples rather than describing the prevalence of FBP in percentage (%) with its CI.

We also estimated the prevalence rates of FBD with 95% CIs by overall and subgroup analysis. In order to do this, the point prevalence rates were first transformed into logit transformed proportions and the transformed data were fitted for a random effects model using DerSimonian-Laird weights [123]. Heterogeneity among the reported prevalence was assessed by computing *p*-values of Cochrane Q-test, τ^2^ and *I*^2^ statics. The prevalence was estimated as the total number of positive samples detected for FBP divided by the total number of sample processed in the laboratory multiplied by 100. Meta-regression analysis was carried out to evaluate a linear relationship between the independent effect size for variables like: the reported bacterial pathogen, region, geographic area, district or town, actual study year, year of publication, sample type and categorized sample size included in the human and environmental sample based FBD studies separately using R package “metafor”. Analysis was conducted using “meta” and “metafor” packages of R programming software [124] version 4.0.3.

The test for heterogeneity (*Q*), the estimate of between-study variance (*τ*^2^), and the estimate for the proportion of the observed variability that reflects the between-study variance (*I*^2^) were used to test and quantify heterogeneity. Heterogeneity chi-square (*Q*-test) and its *p*-value serve as a test of significance to address the null hypothesis. The *τ*^2^ reflects the amount of true heterogeneity on an absolute scale [34], i.e., the total amount of systematic differences in effects across studies. The total variance of a study is the summary of the between and within-study variance and is used to assign weights under the random-effects model. The I^*2*^ is roughly the ratio of between-study variance to the observed variance and used to compare estimates of heterogeneity across meta-analyses. Its values range from 0 to 100%. I^*2*^ = 0%, it means that all of the heterogeneity is caused by sampling error and there is nothing to explain; I^*2*^ = 100%, the overall heterogeneity can be accounted for by the true differences between studies exclusively [35].

## Results

### Systematic review

A simple summary reports with the prevalence of *Salmonella* and pathogenic *E. coli* in human stool and environmental samples were performed using descriptive statistics. In this regard, the current review showed, pathogenic *E. coli* and *Salmonella* have been detected in human stool at different level of occurrence in different parts of Ethiopia, ranging from 0 to 48.34% (*E. coli*), and 41.38% (*Salmonella*). In the present analysis, for the human stool based study, studies from six regional states and the two city councils were included but unfortunately studies conducted in Benishangul Gumuz, Somali and Afar regional states were not screened and not included. Geographic area coverage of the study also indicated, less attention is given to *E. coli* where about 60% of the articles screened for the current systematic review of FBP in the human case consisted of studies that have been conducted on *Salmonella*. Majority of the studies reported prevalence of FBP using routine culture and biochemical tests and none of the study used molecular diagnostic techniques for detection of pathogenic *E. coli* in human stool. In both pathogens, the prevalence in healthcare facility based studies was higher than in the community or non-healthcare facility institution based studies (Table 2).

Environmental samples used in the calculation of the pooled prevalence estimates of FBD in the environment were: Animal sourced foods (ASF), water, RTE foods, and swab from food and human contact surfaces. We found that the two FBP were not equally studied and reported, where majority of the reviewed studies were conducted on *E. coli* than *Salmonella* reporting studies. There was a wide range in prevalence of *E. coli,* from a minimum of 0.35% to the maximum of 81.3% in carcass and retail meat contact surfaces, and in large animal feces, respectively. The prevalence of *Salmonella* ranged from 0 to 57.5%, sequentially in drinking water and other RTE foods (Table 3).

**Fig. 3:**
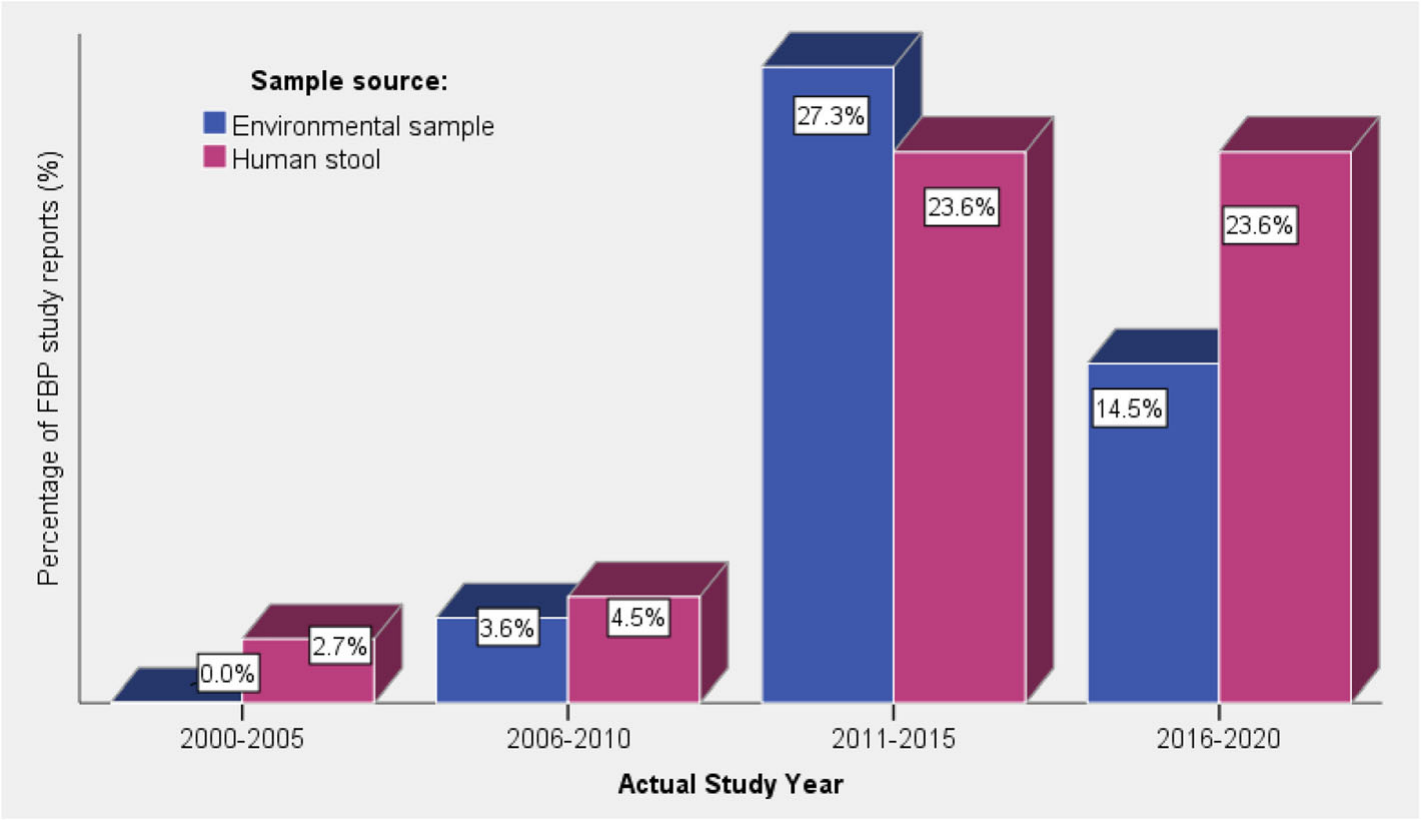
Proportion of reviewed *Salmonella* and pathogenic *E. coli* study reports by actual year of study

Variation in actual study year (actual data collection and laboratory analysis period) of *Salmonella* and pathogenic *E. coli* in Ethiopia from the year 2000 to 2020 was analysed (Table 3). Overall about 50% of the FBP studies included in this review were carried out during the period from 2011 to 2015 during which the environmental sample based FBP studies were the prevailing ones. Nevertheless, during the period from 2000 to 2010 and 2016 to 2020 the number of studies conducted on the FBP in human (stool sample) is higher than studies conducted on environmental samples (Fig. 3). Furthermore, the current review indicates there was up to six years period delay in publications from the actual study period, in such case it may be difficult to estimate occurrence rate of FBD based on year of publication of the articles.

## Meta-analysis

A total of 34,747 study participants and 22,113 environmental samples from 120 manuscripts were analysed to calculate the overall pooled prevalence of FBD/FBP from reports of 20 years on four FBP: *Salmonella*, pathogenic *E. coli, Shigella* and *Campylobacter spp.*, data in Ethiopia. In the absence of subgroups, human and environmental samples were analyzed separately and then merged to estimate overall pooled prevalence. The overall pooled prevalence estimate of FBD from the random effect meta-analysis model was, 8% with 95% CI: 6.5–8.7. Accordingly, the pooled prevalence estimates of FBD is statistically higher (*P* <  0.01) in the environmental samples (11%; 95% CI: 8.8–14.1) than in human stool (6%; 95% CI: 5–6.9) (Table 4). The calculated Cochran’s Q value (χ^2^ (45) =5316, *p* <  0.01) indicated the presence of significant true heterogeneity between human and environmental sample analysis of FBD in Ethiopia.

**Table 4:** Overall pooled prevalence estimates of FBD/FBP in human stool and environmental samples, estimated from a 20 years studies on *Salmonella*, *E. coli, Shigella* and *Campylobacter,* in Ethiopia

Sample source	Number (K)	Prevalence (95%CI)	τ^**2**^	I^**2**^	df	Q (***P*** value)
Human	140	6(5.00,6.93)	0.89	93%	139	1991(< 0.01)
Environment	91	11(8.84,14.1)	1.50	96.7%	90	2745(< 0.01)
**Overall**	**231**	**8 (6.51;8.71)**	**1.31**	**95.7%**	**230**	**5316(< 0.01)**

Q = Heterogeneity chi-square, I^2^ = Variance in effect size attributable to heterogeneity, τ^2^ = Estimate of between study variance, df = degree of freedom

### Subgroup meta-analysis of FBP

In the current meta-analysis, overall 36,002 samples (17,729 human stools and 18,273 environmental samples) were extracted from the 94 included *Salmonella* and pathogenic *E. coli* studies. Subgroup analyses were performed by splitting studies within categorical moderators into subgroups such as sample sources (as human stool and environmental samples), bacterial pathogens tested (*Salmonella* and *E. coli*), administrative regional states of the country, year of study and other variables considered in our study designs (Fig. 2). However, as described under the methodology section and screening procedures, the data on *Shigella* and *Campylobacter* spp*.* are excluded from the subgroup meta-analysis. Separate subgroup analysis of *Salmonella* and *E. coli* indicated a significantly higher prevalence estimate in environmental samples (10.5%; 95% CI: 8.1–13.5) than in human stool (5%; 95% CI: 3.7–6.5) (Table 5). In the presence of subgroups, the estimate of the summary proportion for all studies can be different than that in the absence of subgroups [34]. This is because different estimates for *τ*^2^ are used in different cases. Borenstein et al. [34] also described, the random effects model is used to combine study effects within each subgroup and presence of significant variation between the effects across the subgroups is tested by the fixed-effect model.

**Table 5:** Subgroup analysis pooled prevalence estimates of *Salmonella* and pathogenic *E. coli* by sample

Subgroup	Number (k)	Prevalence (95% CI)	τ^**2**^	I^**2**^	Q(***P*** value)
**Human stool**	67	5(3.7–6.5)	**1.3**	94%	1113(< 0.01)
**Environmental sample**	69	10.5(8.1–13.5)	**1.3**	96.5%	1924 (0.00)
Overall	**136**	**7.4(6.1**–**8.9)**	**1.3**	**96%**	**3185(0.00)**

**Fig. 4:**
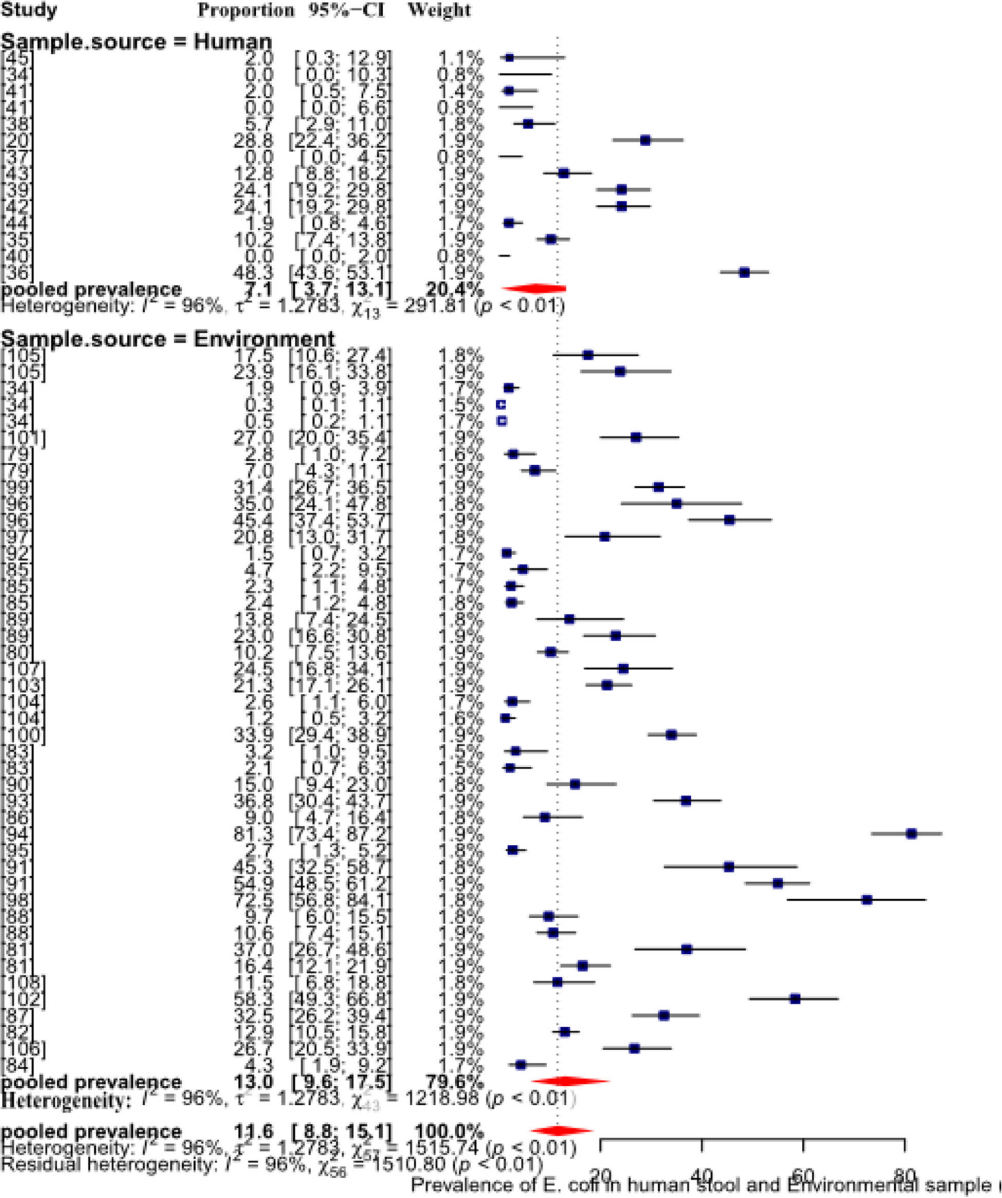
Forest plot of pooled prevalence estimates of *E. coli* in human stool and environmental samples, in Ethiopia

**Fig. 5:**
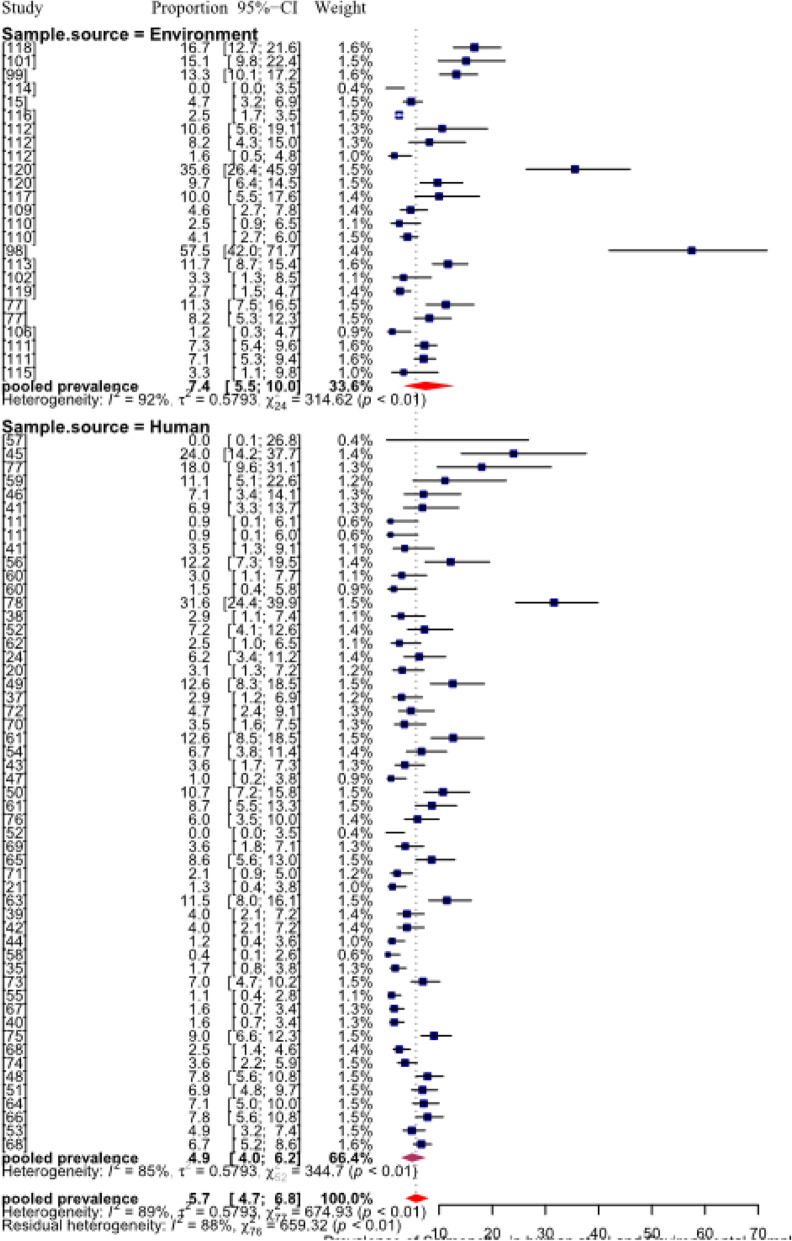
Forest plot pooled prevalence estimates of *Salmonella* in human stool and environmental samples, in Ethiopia

The I^2^ revealed that 96% of the variation is attributable to real heterogeneity (Table 5). Substantial variation between-study was, thus, evident and further subgroup analyses and meta-regression were used to identify sources of heterogeneity. The pooled prevalence of *E. coli* was higher (*p* <  0.05) than that of the *Salmonella* both in the environmental (13%) and human stool (7%) samples. The results also depicted that pathogenic *E. coli* and *Salmonella* contributed for 11.6% (95% CI: 8.8–15.1) and 5.7% (95% CI: 4.7–6.8) respectively, for the overall pooled prevalence estimates of FBD in Ethiopia (Figs. 4; 5).

### Epidemiological factor related subgroup analyses

To determine *Salmonella* and pathogenic *E. coli* prevalence variations under different settings in Ethiopia, separate subgroup analysis was conducted for each principal study variable considered in this review (Fig. 2). Regarding administrative regional states or city council, the result of human stool samples depicted that significantly the highest prevalence (8%, *p* <  0.01) was reported from Addis Ababa and followed by Dire Dawa and Harari regional state. The significantly highest prevalence of FBP (6.4%; 95% CI: 4.1–8.9) was reported in UFC age groups with the highest occurrence rate during the period between 2011 and 2015. Regarding diagnostic techniques, majority of the FBP was diagnosed by routine culture plate, biochemical and agglutination tests (surface antigen detection) (Table 6).

**Table 6:** Subgroup analysis of prevalence estimates of *Salmonella* and pathogenic *E. coli* strains in human stool and the associated risk factors in Ethiopia

Risk factors	Subgroup	Prevalence (95%CI)	τ^2^	I^2^	Q (*P* value)
Age group	Under five children (UFC)	6.4(4.1–8.9)	1.10	95.8%	665.4(< 0.01)
	under 15	4.7(2–10.6)	1.10	81.7%	32.8(< 0.01)
	Adult	4.2(2.6–6.7)	1.10	78.3%	87.4(< 0.01)
	All age group	4.2(2.1–8.1)	1.10	92.7%	137.2(< 0.01)
Stool condition	Diarrheic	5.6(3.9–7.5)	1.20	94.8%	840.1(< 0.01)
	Unidentified	7(2.8–16.1)	1.20	96.0%	125.9(< 0.01)
	Non-diarrheic	3.3 (1.8–5.8)	1.20	76.5%	63.7(< 0.01)
Administrative regional states or city council	Amhara	4.2(2.4–7.1)	1.19	97.1%	592.6(< 0.01)
	Oromia	5.5(2.5–9.2)	1.19	80.9%	52.5(< 0.01)
	SNNP	3.8(2.1–6.7)	1.19	78.0%	68.3(< 0.01)
	Addis Ababa	8.1(4.4–14.2)	1.19	93.7%	189.9(< 0.01)
	Dire Dawa and Harari	6.6(2.6–15.9)	1.19	84.8%	26.3(< 0.01)
	Others	3.4(1–11.1)	1.19	80.7%	15.5(< 0.01)
Geographic area	Northwest Ethiopia	3.6(2–6.4)	1.22	97.3%	559.9(< 0.01)
	Central Ethiopia	7.6(4.6–12.1)	1.22	92.8%	264.8(< 0.01)
	Southern Ethiopia	3.4(1.6–5.5)	1.22	80.0%	70.1(< 0.01)
	Eastern Ethiopia	6.6(2.5–16.2)	1.22	84.8%	26.3(< 0.01)
	Southwest Ethiopia	5.7(2.4–12.9)	1.22	79.2%	28.8(< 0.01)
	Others parts of Ethiopia	4.9(1.6–14.3)	1.22	76.4%	12.7(< 0.01)
Study area (town or district)	Northwest eth areas	3.8(1.3–10.6)	1.05	87.8%	32.8(< 0.01)
	Other	4.7(1.4–8.5)	1.05	82.8%	0.3(< 0.01)
	Hawassa	2.1(0.9–4.5)	1.05	47.0%	15.1(< 0.06)
	Addis Ababa	8.1(4.6–13.8)	1.05	93.7%	189.9(< 0.01)
	Central Ethiopia areas	6.6(2.4–17.1)	1.05	95.4%	65.1(< 0.01)
	East Ethiopia towns	6.6(2.7–15.2)	1.05	84.8%	26.3(< 0.01)
	Gondar	1.1(0.4–3.2)	1.05	0.0%	3.9(< 0.43)
	Bahir Dar	7.7(3.4–16.2)	1.05	98.6%	359.2(< 0.01)
	Jimma	5.8(2.6–12.4)	1.05	79.2%	28.8(< 0.01)
	South Ethiopia area town	7(3.3–14.3)	1.05	71.8%	21.3(< 0.01)
Publication year	2016–2020	4.8(3.3–6.5)	1.22	90.2%	450.2(< 0.01)
	2011–2015	6.1(3.6–10.2)	1.22	97.2%	573.8(< 0.01)
	2000–2010	3.9(1.4–10.5)	1.22	84.7%	26.2(< 0.01)
Actual study year	2011–2015	7.3(4.9–10.8)	1.07	95.6%	594.3(< 0.01)
	2016–2020	4.2(2.8–6.1)	1.07	87.8%	236.9(< 0.01)
	2006–2010	2.9(1.2–6.9)	1.07	90.3%	51.5(< 0.01)
	2000–2005	5.1(1.7–14.2)	1.07	77.5%	13.4(< 0.01)
Study design	HFB R-s	7.9(2.7–20.4)	1.15	91.8%	48.6(< 0.01)
	HFB CS-s	5.6(3.9–7.5)	1.15	94.9%	880.1(< 0.01)
	IB Cs-s	3.6(2–6.5)	1.15	80.6%	61.8(< 0.01)
	CB Cs-s	2.2(0.5–9)	1.15	69.4%	6.50.04
Diagnostic technique	C–Gs and Bt	4.7(3.3–6.3)	1.26	91.8%	575.6(< 0.01)
	C– Bt and ser	5.9(3.4–9.9)	1.26	96.7%	510.4(< 0.01)
	Molecular	6.3(0.7–39.7	–	–	–
Sample size	≤100	7.9(3–18.9)	1.32	69.4%	19.6(< 0.01)
	101–200	5.1(3.2–7.9)	1.32	89.1%	229.8(< 0.01)
	201–300	4.4(2.5–7.6)	1.32	92.7%	220.2(< 0.01)
	301–400	2.8(1.2–6.3)	1.32	89.9%	69.6(< 0.01)
	> 400	7.5(3.1–13)	1.32	98.5%	525.5(< 0.01)

*UFC* under five children, *HFB CS* Health facility based cross sectional study, *CB CS* community based cross sectional study, *IB CS* Institution based cross sectional study, *HFB ret.* health facility based retrospective study, mixed = stool samples collected from both Diarrheic and Nondiarrheic participants, *SNNP* Southern Nations, Nationalities, and Peoples

The separate meta-analysis of various risk factors associated with occurrence of FBP indicates that statistically there are differences in occurrence of FBP among different environmental samples, in Ethiopia. The highest prevalence of *Salmonella* and pathogenic strain of *E. coli*, 36.1% (95% CI: 17.4–60.2), was reported in RTE foods. The study also indicated these FBP are more prevalent in Amhara regional state than in the other regions of Ethiopia. The environmental samples based subgroup analysis also indicated the *Salmonella* and *E. coli* pathogens are by far important in Northwest parts of Ethiopia than in the other study areas or districts included in this review. *Salmonella* and *E. coli* were also found important FBP in ASF with the prevalence estimates of 7.4% (95% CI: 4.5–11.8) and 3.8% (95% CI: 1.9–7.6) in meat (carcass) and its contact surfaces respectively. On the other hand, the occurrence of FBP attributed to poultry products and its contact surfaces, and animal feces was sequentially, 12.8% (CI: 5.6–26.4) and 10.9% (CI: 5.1–22.0) (Table 7).

**Table 7:** Subgroup analysis of prevalence estimates of *Salmonella* and *E. coli* in environmental samples in Ethiopia

Risk factors	Subgroup variables	Prevalence (95% CI)	τ^2^	I^2^	Q (*P* value)
Administrative regional states or city council	Oromia	13.6(8.6–20.7)	1.34	96.1%	539.1(< 0.01)
	Somali	10(4.2–21.9)	1.34	95.9%	147.2(< 0.01)
	SNNP	8.2(3.3–19.1)	1.34	96.2%	131.6(< 0.01)
	Amhara	20(11.4–32.7)	1.34	96.5%	341.2(< 0.01)
	Addis Ababa	6.2(2.8–13.2)	1.34	93.5%	107(< 0.01)
	Others	5.1(2.7–9.5)	1.34	96.8%	369.3(< 0.01)
Geographic area	Southwest Ethiopia	8.9(4.5–16.8)	1.10	91.3%	91.6(< 0.01)
	Central Ethiopia	6.5(4.3–9.7)	1.10	95.0%	477.1(< 0.01)
	Eastern Ethiopia	14.8(7.8–26.5)	1.10	95.3%	168.9(< 0.01)
	Southern Ethiopia	8.3(3.7–17.8)	1.10	96.2%	131.6(< 0.01)
	Northwest Ethiopia	18.3(10.2–30.8)	1.10	93.6%	140.6(< 0.01)
	Northern Ethiopia	15.7(7.2–30.9)	1.10	98.1%	256.5(< 0.01)
	Other	29.7(11.9–57)	1.10	92.9%	42.5(< 0.01)
Study area or town /district	Jimma	8.9(4.5–16.9)	1.13	91.3%	91.6(< 0.01)
	Jigjiga areas	10.2(4.7–20.9)	1.13	95.9%	147.2(< 0.01)
	East shoa towns	9(4.4–17.5)	1.13	84.0%	43.7(< 0.01)
	South eth areas	7.1(2.8–16.8)	1.13	97.0%	131.4(< 0.01)
	Gondar	11.7(5.7–22.3)	1.13	87.6%	56.4(< 0.01)
	Northwest Ethiopia area districts	41.2(21–64.9)	1.13	97.6%	165.6(< 0.01)
	Addis Ababa	6.2(3–12.4)	1.13	93.5%	107(< 0.01)
	Other	11(6.9–17)	1.13	97.0%	602.1(< 0.01)
years of publication	2016–2020	9.2(6.7–12.6)	1.45	96.6%	1432.3(< 0.01)
	2011–2015	15.4(9.2–24.8)	1.45	96.6%	466.2(< 0.01)
	2006–2010	7.8(1.4–33.9)	1.45	95.3%	21.4(< 0.01)
Actual study year	2016–2020	17.4(11.3–25.9)	1.39	95.5%	467(< 0.01)
	2011–2015	8.1(5.7–11.4)	1.39	96.8%	1294(< 0.01)
	2006–2010	7.8(2.8–19.8)	1.39	93.9%	65.9(< 0.01)
Sample size	≤ 100	22(14–32.8)	1.13	89.7%	146.3(< 0.01)
	101–200	11.6(7.6–17.4)	1.13	95.9%	507.9(< 0.01)
	201–300	9.8(5.5–17)	1.13	97.0%	369.2(< 0.01)
	301–400	9.6(4.9–17.8)	1.13	96.7%	241.2(< 0.01)
	>400	3(1.5–5.7)	1.13	94.6%	167(< 0.01)
Sample type	Meat or carcass contact surfaces	3.8(1.9–7.6)	1.20	90.0%	90(< 0.01)
	Meat or carcass	7.4(4.5–11.8)	1.20	91.9%	223.5(< 0.01)
	Animal feces	10.9(5.1–22)	1.20	98.7%	460.4(< 0.01)
	Raw milk	22.8(12–39.1)	1.20	94.8%	133.6(< 0.01)
	RTE food	36.1(17.4–60.2)	1.20	95.2%	83(< 0.01)
	Food and hand contacting surfaces	7.5(2.9–18)	1.20	94.5%	73.3(< 0.01)
	Dairy products	15.3(5.6–35.8)	1.20	92.9%	42.5(< 0.01)
	Drinking water	1.4(0.2–9.7)	1.20	68.5%	3.2(< 0.01)
	River water	38.8(11.5–75.6)	1.20	95.7%	23.4(< 0.01)
	Wastewater	11.5(1.3–56.0)	–	–	0(< 0.01)
	Poultry products & contact surfaces	12.8(5.63–26.4)	1.20	96.5%	144.5(< 0.01)

### Meta-regression

We observed that the heterogeneity between FBP in the human and environmental samples is high when studies are evaluated overall (I^2^ = 96%; τ^2^ = 1.3 *P* = 0.00) (Table 5). Hence, sample size was used both as a discrete variable and binned into categories in meta-regression analysis to explore the main factors influencing prevalence estimation and sources of heterogeneity. In line with this, meta-regression analysis was performed on overall prevalence estimates of FBD as well as separately for each variable included in the human and environmental sample based FBD estimates. Meta-regression analyses, revealed that the overall prevalence estimates were not significantly modified by the actual study year and district or town of the studies (*p* > 0.05). However, administrative regional state, geographic area, FBP involved (*Salmonella* or *E. coli*), year of publication, source of sample (from human or environment) and categorized sample size significantly affected the estimation of point prevalence (*P* <  0.05) (Table 8).

**Table 8:** Meta-regression analysis of risk factor associated with pooled prevalence estimates of FBP in Ethiopia

Covariate	Coefficient	*P* value	95% CI
Region	−0.11	< 0.01**	−0.19– (−0.03)
Geographic area	0.16	0.01**	0.03–0.29
Publication Year	0.46	0.02*	0.06–0.86
Sample source	0.52	0.04*	0.04–1.01
Bacterial Pathogen	0.32	0.01**	0.07–0.57
Actual Study Year	0.09	0.55	−0.22–0.41
District town	−0.01	0.56	−0.04 – 0.02
Sample size	−0.23	< 0.01**	−0.40–(−0.07)

Note: *= Significant, **= highly significant

The significant variables (*p* <  0.05) are then subjected to multivariate meta-regression analysis. The multivariate meta-regression analyses, however, indicated that only regional administrative state labeled as others, FBP *Salmonella*, stool sample and sample size less than one hundred were statistically associated with the pooled prevalence estimates of FBP (Table 9).

**Table 9:** Multivariate meta-regression results of risk factor associated with pooled prevalence estimates of FBP, in Ethiopia

Covariate		Coefficient	*P* value	95% CI
Administrative regional states or city council	Addis Ababa	Reference	–	–
	Amhara	0.27	0.77	−1.52–2.06
	Dire Dawa and Harari	−1.13	0.3	−3.28–1.02
	Oromia	0.03	0.95	−0.84–0.89
	SNNP	0.36	0.65	−1.18– 1.90
	Somali	−1.36	0.2	−3.43– 0.71
	Tigray	−1.25	0.33	−3.79–1.29
	Others	−1.37	<.01**	−2.36–(−0.37)
	Central Ethiopia	Reference	–	–
Geographic area	Eastern Ethiopia	1.20	0.19	−0.59– 3.00
	Northern Ethiopia	0.97	0.39	−1.23–3.16
	Northwest Ethiopia	−0.60	0.51	−2.40 –1.19
	Southern Ethiopia	−0.86	0.26	−2.36–0.63
	Southwest Ethiopia	−0.22	0.67	−1.23–0.79
	Others	0.99	0.08	−0.10–2.09
Bacterial genus	*E. coli*	Reference	–	–
	*Salmonella*	−0.76	<.01**	−1.25–(− 0.26)
Sample source	Environment	Reference	–	–
	Human (stool)	−0.52	0.05*	−1.05–(−0.01)
	> 400	Reference	–	–
Sample size	301–400	0.40	0.37	−0.47–1.28
	201–300	0.25	0.51	−0.50–0.99
	101–200	0.48	0.19	−0.24–1.19
	≤100	1.26	<.01**	0.45–2.07
Publication Year	2000–2010	Reference	–	–
	2011–2015	0.43	0.46	−0.72–1.58
	2016–2020	0.03	0.96	−0.08–1.13

Note: * = Significant, ** = highly significant

Meta-regression analysis was also performed separately for each variable included in human and environmental sample based FBP estimates. The predictors included in the environmental sample sources analysis were administrative regional states, geographic area, sample type, publication year, bacterial genera, actual study year, specific study area (district) and categorized sample size. Furthermore, patient age group, sample type, study design and diagnostic technique used for the study were also included in the univariate meta-regression on FBP estimates from human sourced samples. The univariate meta- regression analysis showed that the prevalence estimate of FBP in human stool was only significantly modified by geographical area of the study (*p* <  0.05). In the case of environmental samples based FBP estimates, actual study year, year of publication and categorized sample size subjected to multivariate meta- regression because they are significant (*P* ≤ 0.05).

The multivariate meta-regression analysis indicated geographic areas northwest and southern Ethiopia had a significant effect on the stool based pooled prevalence estimates of FBP. On the other hand, actual study year during the period 2011–2015 and categorized sample sizes of ≤100,101–200, and 201–300 were significantly associated (*p* <  0.05) with the environmental sample based pooled prevalence estimates of FBP (Table 10).

**Table 10:** Multivariate meta regression results of separate human stool and Environmental sample based proportion estimates of FBP in Ethiopia

Covariate Variables			Coefficient	***P*** value	95% CI
FBP in the human stool	Geographic area	Central Ethiopia	Reference	–	–
		Eastern Ethiopia	−0.15	0.76	−1.20,0.90
		Northwest Ethiopia	−0.76	0.04*	−1.51, 0.02
		Southern Ethiopia	−0.97	0.01**	−1.74,0.20
		Southwest Ethiopia	−0.30	0.55	−1.27,0.67
		Others	−0.45	0.46	−1.65, 0.74
FBP in the environmental samples	Sample size	> 400	Reference	–	–
		301–400	1.27	0.04*	−0.07,2.47
		201–300	1.21	0.04*	−0.08,2.35
		101–200	1.52	<.01**	0.48,2.55
		≤ 100	1.75	<.01**	0.64,2.85
	Actual study year	2016–2020	Reference	–	–
		2011–2015	−1.12	< 0.01**	−1.87,-0.36
		2006–2010	−1.18	0.22	−3.05, 0.70
	Year of publication	2011–2015	Reference	–	–
		2016–2020	−0.06	0.15	−1.40,0.21

Note: * = Significant, ** = highly significant

## Discussion

### Overall foodborne pathogen (FBP) estimates in Ethiopia

Foodborne diseases (FBD) have been an issue for all societies since the beginning of humanity. However, the types, severity and impacts of these illnesses have changed through the ages and are still diverse across regions, countries and communities [125]. Based on 120 studies carried out between 2000 and 2020 in nine administrative states and two city councils in Ethiopia, this review identified that pathogenic *E. coli, Salmonella, shigella, Campylobacter spp.* are common FBP as was reported in human stool, animal sourced foods, other non-animal sourced foods and contact surfaces related with foods. The current meta-analysis indicated, the overall pooled prevalence estimate of FBP, in Ethiopia, is 8%; 95% CI: 6.5–8.7 with statistically higher (*P* <  0.01) occurrence rate in the environmental samples (11%; 95% CI: 8.8–14.1) than in human (6%; 5–6.9) (Table 4). The higher prevalence in the environmental samples including ASF indicates the level of sanitation in the country. Havelaar [126], also reported that occurrence of FBD is higher in developing countries as it is closely linked to poverty. WHO Foodborne Epidemiology Reference Group (FERG) also estimated about 35% of the global burden of FBD is caused by ASF [126].

The overall prevalence estimate of *Salmonella* and pathogenic *E. coli* calculated from 94 studies was 7.4% (95% CI: 6.1–8.9) and I^2^ revealed that 96% of the variation is attributable to real heterogeneity (Table 5). Meta-regression analyses also revealed that majority of the risk factor like the FBD causative pathogen, sample source, administrative regional state, geographical area, and sample size and publication year significantly modify (*P* <  0.05) the overall prevalence estimates of *Salmonella* and *E. coli* in Ethiopia (Tables 8 and 9). The separate subgroup meta-analysis of studies conducted, from the year 2000 to 2020, also depicted that *Salmonella* and *E. coli,* sequentially accounted for 5.7% (95% CI: 4.7–6.8) and 11.6%; (8.8–15.1) of the overall pooled prevalence of FBD in the country. The overall average prevalence of 34.2% FBD previously reported in seven African countries [6] is much higher than the current pooled prevalence estimates. Prevalence differences may be due to the differences in number of FBP included in the review for FBD prevalence estimate, in our case studies on four FBP.

Moreover, Enteropathogenic bacteria like the genus *Salmonella* and pathogenic *E. coli* (mainly enteropathogenic *E. coli* (EPEC), enterohemorrhagic *E. coli (*EHEC) and enteroinvasive *E. coli (*EIEC) are widespread and important causes of foodborne infections in human, particularly in developing countries including African countries. This may be due to difficulties in securing optimal hygienic food handling practices [7, 127]. In addition to this, most known human infectious diseases and approximately three quarters of newly emerging infections come from animals [12, 23, 128]. *Salmonella*, specifically S. Typhimurium and S. Enteritidis, are the commonest serotypes causing human infection, and are frequently detected in farm animals as are other serotypes known to be human pathogens [129, 130]. Eggs and poultry products have been described as the main vehicles for the transmission of human salmonellosis [131, 132]. To reduce this high FBD prevalence, a key challenge is to adopt approaches that have been proven successful in high-income countries to low- and middle-income countries (LMIC) in an economically and culturally acceptable way [126].

On the other hand, the present systematic review indicated that there has been a delay period up to six years between the date of publications and the actual study period [28, 59, 77]. This may be a source of time-window bias in estimating occurrence rate of FBD at regional or national level based on year of publication of the articles. Few systematic review and meta-analysis have been conducted on FBP in Ethiopia and existing reports only assessed evidence of publication bias [26, 133] and did not report the actual study period as source of bias. Hence, research results need to be published as soon as the study is finalized. This helps to minimize such bias and to disseminate up-to-date information to the stakeholders.

### The FBP, Salmonella and pathogenic *E. coli*, in human stool

The pooled prevalence of *Salmonella* and *E. coli* was estimated from 51 studies conducted, on an aetiological isolation or detection, in stool samples collected from inpatient, outpatient and community based study designs. During the period between 2000 and 2020, the pathogens had been occurring at a pooled prevalence of 5%; 95% CI: 3.7–6.5(Table 5), though the prevalence variation between *Salmonella* and *E. coli* and their combined occurrence rate variation in different epidemiological risk factors, in Ethiopia is evidenced (Table 6). In line with this, the pooled prevalence of pathogenic pathogenic *E. coli* in human stool is higher (7, 95% CI: 3.7–13.1) than that of the *Salmonella* (5%; 95% CI: 4.0–6.2) (Figs. 4; 5). The variations of FBP prevalence in human, animal and environmental samples might be attributed to the level of infection in animal-human and or contamination of foods in the country. Because, *Salmonella* and *E. coli,* have a predilection limited to the digestive tracts of both humans and animals hosts and their presence in other habitats such as water, environment, and food represents fecal contamination [134, 135]. In Ethiopia, several factors including undernutrition (malnutrition), HIV-AIDS, the unhygienic living circumstances and the close relations between humans and animals may substantially contribute to the occurrence of salmonellosis [27, 29] and *E. coli* infection [39, 73, 83, 136]. Unequal number of reports used might also be a source of the variation between the pathogens, where higher number of studies conducted on *Salmonella* spp. is eligibly screened than studies on *E. coli* for the present review.

Of the nine administrative regional states (considering Sidama region in SNNP) and two city councils in Ethiopia, studies conducted in a six regional states and the two city councils depicted that significantly the highest prevalence was reported from Addis Ababa, and followed by combined estimates from Dire Dawa and Harari regional state. Studies conducted in Tigray and Gambella regional states designated as “others” in subgroup analysis showed the least prevalence estimates of *Salmonella* and *E. coli*. The analysis also showed variation of FBP prevalence among geographic and specific study area or towns included. From this prospective, *Salmonella* and *E. coli* were more important in central, eastern and southwest Ethiopia with 7.6, 6.6 and 5.7% pooled prevalence, respectively. However, the meta-regression analysis depicted that the pooled prevalence of the FBP is statistically varied (*P* <  0.05) with the results of studies conducted in northwest and southern in Ethiopia (Table 10). With regard to specific study area, the highest and the lowest prevalence were reported from Bahir Dar and Gondar towns of Amhara regional state, respectively (Table 6).

The current pooled prevalence estimate of *Salmonella* and pathogenic *E. coli* in human stool*,* 5%, is comparable with health facility based cross sectional study findings reported by Eguale et al. [29] and Beyene and Tasew [57], who report a 6.2% *Salmonella* prevalence from Addis Ababa and Jimma, respectively. Another previous study conducted in Addis Ababa, however, reported higher prevalence of diarrheagenic bacterial pathogens than the current prevalence estimates [41]. Higher prevalence up to 13.8% in *E. coli* was also reported from Debre Markos [48]. On the other hand, lower prevalence of *E. coli* [41, 44] and *Salmonella* [41] were previously reported from Hawassa and Debre Markos, respectively. The observed heterogeneity in prevalence of these pathogens among regions or geographic areas may be associated with differences between experience and awareness of hygienic measures of people living in the regions and districts or towns. The articles used in this review did not cover all geographic areas and there is also a factual difference in study reports between urban and rural areas of the region which might be another factor for the variation. On the other hand, as there is no previous meta-analysis report of FBP in human stool from Ethiopia, there is a data gap for comparison of the pooled prevalence.

Enteric bacterial pathogens including DEC and NTS, and their products, are the major causes of acute diarrhea [7, 136]. Likewise, we found a variation in the prevalence estimates of FBP among the stool sample conditions diarrheic, non-diarrheic, and unidentified stool considered in this analysis. The least prevalence value, 3.3%(1.8–5.8), was found in non-diarrheic patients. The review also revealed that pooled prevalence of *Salmonella* and *E. coli* varies among different age groups (Table 6). Significantly the highest prevalence, (6.4%; 95% CI: 4.1–8.9) was recorded in UFC age groups with the utmost occurrence rate during the period between 2011 and 2015. The higher prevalence during this period might be connected with the fact that majority of the studies included in this review were conducted between 2011 and 2020 years. Reduction in prevalence during 2016 and 2020 period, however, might implies presence of little public awareness about FBP to the level that may not undermined. The prevalence estimates in under 15, adults and all age group categories were 4.7%; 95% CI: 2–10.6, 4.2%; 95% CI: 2.6–6.7 and 4.2%; 95% CI: 2.1–8.1, respectively.

Similarly, variation in prevalence and geographical distribution of FBP among age group categories at different geographic areas has also previously reported by Ayana et al [137].

Comparable to this study, 6.5% *Salmonella* prevalence was reported in UFC in Addis Ababa, and the higher proportion of FBD in UFC might be associated with lower immune status of younger children [71]. *Salmonella* infection, particularly NTS usually causes self-limiting gastroenteritis characterized by diarrhea, abdominal pain and vomiting in people of all ages [29, 129]. Drinking untreated water from private wells and recreation in surface waters are also risk factors for FBD, like sporadic *Salmonellosis* in children [138]. Intrinsic risk factors can also influence occurrence of NTS and diarrheagenic *E. coli*. Gastric hypoacidity in infants, due to pernicious anemia, or caused by antacids and H-2 blockers could predispose individuals to *Salmonellosis* [139]. Poor environmental conditions, socioeconomic status and behavioral factors are also strongly associated with the risk of diarrheal disease transmission. In Ethiopia about 13% of children under age of 5 years had diarrhea and according to the World Health Statistics 2011, 27% deaths of UFC in Ethiopia is due to diarrheal diseases [140].

Apart from Ethiopia, Harb et al. [132] also mentioned that prevalence of FBP like *Salmonella* is usually higher in children under the age of five and rates of contamination vary between countries due to a number of factors including the source and type of sample. Another study also reported that diarrheal diseases are one of the most important causes of illness in young children in developing countries. It contributes about 24–30 and 25% deaths, in infant and among children aged between 1 and 4 years [141]. The difference in *Salmonella* prevalence among humans is dynamic in nature, and it is not surprising to capture variations between countries. Such variations might be attributed to several factors impacting NTS levels in food and water, which play a major role in human exposure to infection [131, 132]. In this study, the highest prevalence was found in studies in which smaller sample was used whereas the least prevalence was reported in studies that analysis a sample size between 300 and 400. However, observed significant estimate in sample size ≤100 categories may not exactly show true effect size because a small studies can produce false-positive results, or small sample size may over-estimate the magnitude of an association [142].

Overall, higher pooled prevalence of *Salmonella* and pathogenic *E. coli* was recorded in studies employing health facility based design than in the community or non-health facility institution based study types (Table 6). Higher FBP prevalence could be because of the fact that sick people admitted to healthcare facilities may have been exposed to at least one pathogen which is probably associated with the development of clinical signs and symptoms, for which the patient is admitted. Majority of the study reports used in this review are conducted using routine culture and biochemical, agglutination tests. None of the studies employed molecular diagnostic techniques for detection of pathogenic *E. coli* in human stool (Table 2). Besides, the routine culture and biochemical test based pathogen detection, in 5.9%; 95% CI: 3.4–9.9 of the studies, anti-sera (agglutination) tests were also used. In 4.7%; 95% CI: 3.3–6.3 studies; however, *Salmonella* and pathogenic *E. coli* were detected only with culture and biochemical tests.

The methods and laboratory protocols used for isolation and identification of the pathogens were variable across studies. Molecular diagnostic technique was not considered for comparison of the diagnostic technique estimates because it is employed only in three studies (Table 6). However, molecular methods like genotypic identification remain the most popular and most reliable techniques recommended for differentiating diarrheagenic strains from nonpathogenic members of the stool flora [143]. It avoids all inconvenience of phenotypic assays, like variation in enzymatic activity when bacteria are cultured in different media, emergence of biochemical mutants and presence of strain of different species that are very closely related and possess the same phenotype but different genotype [144]. The molecular characteristics of organisms provide markers for investigation of outbreaks, attribution studies, and assessment of potential virulence or epidemic potential [143].

The usual use of routine culture and biochemical tests in disease diagnosis, however, implies little attention is given to FBD detection which may be partially due to limited access to advanced laboratory facility in the country. Moreover, limited access to advanced facilities hinders the validity and depth characterization of pathogens. Available studies also suggested that, in Ethiopia, little is known about public health effect of bacterial pathogens like *E. coli* as FBP due to lack of a well-documented system and an integrated surveillance [145]. A comprehensive investigation approach is therefore needed as input towards the achievement of evidence based prevention and mitigation strategies.

### The FBP, Salmonella and pathogenic *E. coli*, in the environment samples

The result of 43 studies screened for the current meta-analysis also identified *E. coli* and *Salmonella* as a commonly reported FBP in different environmental samples in Ethiopia. The systematic review indicated the highest prevalence of *E. coli* was 81.3% (73.43–87.25) reported in a study by Hussen [96] from northern Ethiopia whereas its correspondence for *Salmonella* was 57.5% (41.96–71.69) investigated in northwest areas of Ethiopia [100]. The lowest prevalence was reported as 0.35% for *E. coli* in carcass from Central Ethiopia [37] and 0% for *Salmonella* [116]. In contrary to the case of FBPs in human, there were a higher number of eligible studies on *E. coli* from environmental samples which also covered wider geographic areas than studies on *Salmonella* spp. (Table 3). Likewise, variation of bacterial FBP in different food items and other environmental samples was reported in previous studies [8, 146].

In agreement with the prevalence in human stool, the highest pooled prevalence of *E. coli* and *Salmonella* were reported in sample size ≤100. Therefore, the variation in sample size and sample type analyzed by different researchers might also attributed to variation in prevalence of FBP. Unlike the human cases, the highest prevalence of *Salmonella* and *E. coli* was reported during the 2016–2020 which may be connected to the growing number of higher education and research institutes in Ethiopia that has been improving the capacity of pathogen detection that minimizes, for instance, false negative cases. On the other hand, the higher prevalence estimates in sample size ≤100 might be due to effects of uncertainty. Larger sample sizes give more reliable results with greater precision and power masking the effects of uncertainty [142]. Likewise, the number of environmental sample based studies included in this reviews from the study period 2016–2020 are less than that included from the study periods between 2011 and 2015.

Overall the environmental sample based pooled prevalence of *Salmonella* and *E. coli* was 10.5% (8.1–13.5) with I^2^ = 96% and Q(p) = 1924(0.00) (Table 5) and the actual study year, publication year and categorized sample size significantly influences (*P* <  0.05) the prevalence estimates (Table 10). The current pooled prevalence in food is partially comparable with reports of different studies [26, 133, 147]. A pooled prevalence value of *E. coli*, 15%, was recently reported by Assefa and Bihon [26] in ASF, from Ethiopia. A 9 and 10% pooled prevalence of *Salmonella* in ASF and poultry meat was also respectively, reported in previous studies [133, 147]. The current pooled prevalence of *Salmonella* and pathogenic *E. coli* in the environmental sample, 10.5% (Table 5), is however, higher than the pooled prevalence of *E. coli* reported in meat and meat products [133].

Among the sample type analyzed, the highest prevalence (*p* <  0.01) was found in RTE foods (i.e. different street vended foods such as bonbolino, sambusa, fuol, cooked potato, RTE vegetables, White lupin (*Lupinus albus*) and etc. which are eaten in the street in Ethiopia). The results also reported the presence of *Salmonella* and *E. coli* in 7.4% (95% CI: 4.5–11.8) and 3.8% (95% CI: 1.9–7.6) of meat (carcass) and its contact surfaces, respectively. On the other hand, the occurrence of FBP attributed to poultry products and its contact surfaces, and animal feces was sequentially, 12.8% (CI: 5.6–26.4) and 10.9% (CI: 5.1–22.0) (Table 7). Other available study, on averaged reported a higher prevalence of *E. coli,* 37.6% in raw foods, and 31.6% in RTE foods. Its corresponding prevalence for *Salmonella* in seven African countries, namely Benin, Botswana, Ghana, Kenya, Nigeria, Sudan and Uganda, was 19.9 and 21.7% [6]. Relative contribution of ASF to FBD burden differs widely between subregions, and between countries within the same subregion [126]. Carcasses from apparently healthy animals are generally assumed to be free of bacterial pathogens but contamination occurs in slaughterhouses. Nevertheless, the occurrence of *E. coli* and *Salmonella spp* in ASF in Ethiopia is high due to many reasons like illegal slaughtering of animals in open fields and unhygienic slaughter practices in the abattoirs [148, 149]. As justified by Tadesse and Gebremedhin [148], the skills of personnel involved in gut evisceration, carcass examination, carcass handling, and the hygienic standards of the slaughterhouses may also result in differing prevalence of the FBP between different studies. Besides, contamination of meat with *Salmonella spp.* in slaughterhouses in Ethiopia is also significantly associated with slaughtered animal species. Salmonella *spp.* prevalence was 3.86, 4.53, 8.34 and 10.76% in goat carcasses, beef carcasses, minced beef and milk respectively [148]. A previous study also reported higher prevalence of *Salmonella* 7.1, 8.4 and 9%, in slaughtered cattle, sheep and goats respectively, in Ethiopia [150].

The pooled prevalence of FBP in the present meta-analysis was 22.8% (12–39.1) in raw milk and 15.3% (5.6–35.8) in other dairy products (Table 7). However, a study by Keba et al. [8], from Ethiopia reported only a 6 and 10% pooled prevalence of *Salmonella* and *E. coli* respectively in raw cow milk. On the other hand, study conducted by Abunna et al. [151] on boiled milk and Bedasa et al. [91] on pasteurized milk reported no *E. coli* positive samples. Similarly, Tesfay et al. [152] and Ejeo et al. [114] did not detect *Salmonella* in pasteurized milk and other processed dairy product samples. This supports the concept that it is not common to detect pathogens like *E. coli* and *Salmonella* in processed dairy products but pathogens can enter the food chain as contaminants during post process product handling up to the consumption time. Specifically, *E. coli* is a marker of fecal contamination and the higher prevalence indicates reduced level of hygienic practice [6, 153]. Paudyal et al. [6], also explained that differences in food types as well as non-uniform protocols for sampling and identification might have contributed to heterogeneity although some high prevalence data could be factual due to an extensive variety of raw and RTE foods. This significant prevalence of FBP in different food items and other environmental samples may be linked with cross contamination of the food items.

A safe drinking water, also called potable water is water does not contain harmful or potentially harmful substances and does not present any risk to human health [154, 155]. However, one study specifically reported 3.3% *Salmonella* prevalence in drinking water [117]. Our study also showed 38.8 and 11.5% combine prevalence estimate of *Salmonella* and pathogenic *E. coli* in river water and wastewater, respectively. The current review findings are partially in agreement with the reports of Desta [156], Tsega et al. [157] and Melaku [154] from Ethiopia, who reported, higher proportions of total coliform and fecal coliform counts above the recommended national and international standards in drinking water. Denno et al. [138] reported that drinking untreated water from private wells and recreation in surface waters are risk factors for FBD. Furthermore, both *Salmonella* and *E. coli* normally live in the intestinal tracts of in healthy and disease conditions which may increase the contamination and cross contamination of different foods and drinking water. The pathogens are also naturally found in the environment and in both domestic and wild animals [6, 146]. Some animal species are asymptomatic and act carriers; hence they play a prominent role in epidemiological distribution of the FBD [79].

In general occurrence of FBP may vary with different risk factors but it is difficult to identify the specific factors that might have contributed to high heterogeneity of FBP prevalence. The prevalence data could be factual due to an extensive variety of foods that are processed or handled under different hygienic conditions. As described in the available studies, most of the FBP are not endogenous contaminants but are introduced as exogenous contaminants during handling, processing and preparation [5, 6]. The separate analysis of different risk factors associated with occurrence of FBP in the environmental samples also indicates significant differences in occurrence of FBD among the administrative regions or city councils, in Ethiopia. Significantly, the highest prevalence (*p* <  0.01) of *Salmonella* and pathogenic *E. coli* is reported from Amhara regional state (Table 7). Such variation could be due to different methods of diagnosis, sample sizes and study locations among other reasons [26]. The least prevalence estimate, 6.5% (4.3–9.7), reported from central Ethiopia, however, might implies presence of relatively better awareness about FBP in and around the capital of the country, Addis Ababa. Lack of regular reports and unequal accessibility of FBD data at different levels due to poor cooperation among human and environmental health professionals, and animal health or veterinarians can also be one reason for the variation among regions and districts. As a matter of fact, there is poor information sharing and communication across the key relevant sectors and multi-sectoral working mechanism in response to disease outbreaks and other one health-related hazards is almost absent in Ethiopia [158].

### Limitations of the study

This study has some limitations which is obviously expected from such a study. Possible sources of bias include the inclusion/exclusion criteria, the chosen database, the date, the language, and the number of articles included as well as the article type selected for this study. Moreover, small number of pathogens considered for the analysis is also a limitation—i.e., only pathogenic *E. coli, Salmonella*, *Shigella* and *Campylobacter* spp., were included in the study to determining the overall pooled prevalence of FBP in Ethiopia. This may have impacts on the statistical estimates of the pooled prevalence of FBP. We believe that the inconstant number of studies in each subgroup variable (Fig. 2) may also affect the results of subgroup analysis as less than ten studies were also included in few subgroups. Besides, most of the included studies detected the FBP using routine culture and biochemical tests. As a result, our study only gives the general picture of FBP in Ethiopia and couldn’t estimate relative importance of each serotype or pathotype.

## Conclusion

In conclusion, overall pooled prevalence estimate of bacterial FBP was 8%, with relatively higher prevalence estimates in the environmental samples than in human stool. There was a substantial variation in occurrence of FBP, specifically *Salmonella* and *E. coli,* under different epidemiological settings like, in different food items, food contact surfaces as well as among age group categories. On the other hand, the current review highlights that FBP are important in all age group categories in Ethiopia though the significantly highest prevalence estimate*,* 6.4%, was reported in UFC.

Furthermore, in the majority of the eligibly included studies in the current review, FBP were identified using routine culture and biochemical test based diagnostic techniques which imply there is a high need for advanced laboratory protocols to enhance the diagnosis and detection of FBD in Ethiopia. Besides, almost all of the retrieved studies were conducted in a fragmented manner either in human, animal or environmental samples which indicates there is a research gap in source attribution of the disease in the country. Thus, further FBD studies at the human, animal and environmental interface employing advanced diagnostic techniques is needed in order to investigate the source attributions of human or animal infection in an integrated one health approach.

## Supplementary Information

**Additional file 1: Annex 1.** Specific key words (search terms) used to retrieve potential articles to identifying the most important FBP in children in Ethiopia.

## Data Availability

The data used in this systematic review and meta-analyses was presented in the study and also the remaining data is accessible by requesting, from the corresponding author. Additionally, as supplementary file Annex 1 is submitted with this paper.

## Abbreviations

ASF: Animal sourced foods
CSA: Central Statistical Agency
EHEC: Enterohemorrhagic *E. coli*
EIEC: Enteroinvasive *E. coli*
EPEC: Enteropathogenic *E. coli*
FBD: Foodborne diseases
FBP: Foodborne pathogens
FERG: Foodborne Epidemiology Reference Group
NTS: Non typhoidal *Salmonella*
PCR: Polymerase chain reaction
RTE food: Ready to eat food
SNNP: Southern Nations, Nationalities, and Peoples
UFC: Under five children
