# Supplementary material for: Prevalence and epidemiological distribution of selected foodborne pathogens in human and different environmental samples in Ethiopia: a systematic review and meta-analysis
Source: One Health Outlook. 2021 Sep 3;3:19. doi: 10.1186/s42522-021-00048-5 (PMC8414678; doi:10.1186/s42522-021-00048-5)
Supplement: Supplementary file 1 — Additional file 1: Annex 1. Specific key words (search terms) used to retrieve potential articles to identifying the most important FBP in children in Ethiopia. [file 42522_2021_48_MOESM1_ESM.docx]

| Annex 1. Search terms used to retrieve potential articles to identifying the important FBP causing diarrhea in Ethiopia |  |
| --- | --- |
| "Foodborne Disease"[All Fields] AND "Ethiopia"[All Fields] AND ("2000/01/01"[PubDate] "2020/07/24"[PubDate]), |  |
| "Foodborne Disease"[All Fields] AND "children"[All Fields] AND "Ethiopia"[All Fields] AND ("2000/01/01"[PubDate] : "2020/07/24"[PubDate]), |  |
| "Foodborne Disease"[All Fields] AND "children"[All Fields] AND "Ethiopia"[All Fields] AND ("2000/01/01"[PubDate] : "2020/07/24"[PubDate]), |  |
| "Foodborne Disease"[All Fields] AND "Epidemiology"[All Fields] AND "Ethiopia"[All Fields] AND ("2000/01/01"[PubDate] : "2020/07/24"[PubDate]), |  |
| "Foodborne Pathogens"[All Fields] AND "children"[All Fields] AND "Diarrhea"[All Fields] AND "Ethiopia"[All Fields] AND ("2000/01/01"[PubDate] : "2020/07/24"[PubDate]), |  |
| “Foodborne Pathogens"[All Fields] AND "Epidemiology"[All Fields] AND "Diarrhea"[All Fields] AND "Ethiopia"[All Fields] AND ("2000/01/01"[PubDate] : "2020/07/24"[PubDate]), |  |
| "Prevalence"[All Fields] AND "Foodborne Pathogens"[All Fields] AND "Diarrhea"[All Fields] AND "Ethiopia"[All Fields] AND ("2000/01/01"[PubDate] : "2020/07/24"[PubDate]), |  |
| "Prevalence"[All Fields] AND "Foodborne Pathogens"[All Fields] AND "Diarrhea"[All Fields] AND "children"[All Fields] AND "Ethiopia"[All Fields] AND ("2000/01/01"[PubDate] : "2020/07/24"[PubDate]), |  |
| “Prevalence"[All Fields] AND "Foodborne Pathogens"[All Fields] AND "Epidemiology"[All Fields] AND "Diarrhea"[All Fields] AND "children"[All Fields] AND "Ethiopia"[All Fields] AND ("2000/01/01"[PubDate] : "2020/07/24"[PubDate]). |  |
